# Further Characterization of Fungal Halogenase RadH and Its Homologs

**DOI:** 10.3390/biom13071081

**Published:** 2023-07-06

**Authors:** GuangRong Peh, Gregory A. Gunawan, Terence Tay, Elaine Tiong, Lee Ling Tan, Shimin Jiang, Yi Ling Goh, Suming Ye, Joel Wong, Christopher J. Brown, Huimin Zhao, Ee Lui Ang, Fong Tian Wong, Yee Hwee Lim

**Affiliations:** 1 Institute of Sustainability for Chemicals, Energy and Environment (ISCE2), Agency for Science, Technology and Research (A*STAR), 8 Biomedical Grove, Neuros #07-01, Singapore 138665, Singapore; peh_guangrong@isce2.a-star.edu.sg (G.P.); gregoryag@u.nus.edu (G.A.G.); goh_yi_ling@isce2.a-star.edu.sg (Y.L.G.); ye_suming@isce2.a-star.edu.sg (S.Y.); joel_wong@g.harvard.edu (J.W.); 2 Institute of Molecular and Cell Biology (IMCB), Agency for Science, Technology and Research (A*STAR), 61 Biopolis Dr, Proteos #07-01, Singapore 138673, Singapore; elaine_tiong@imcb.a-star.edu.sg (E.T.); tan_lee_ling@imcb.a-star.edu.sg (L.L.T.); 3 Singapore Institute of Food and Biotechnology Innovation (SIFBI), Agency for Science, Technology and Research (A*STAR), 31 Biopolis Way, Nanos #01-02, Singapore 138669, Singapore; songbuck.tay@gmail.com (T.T.); zhao5@illinois.edu (H.Z.); 4 Disease Intervention Technology Laboratory, Institute of Molecular and Cellular Biology, Agency for Science, Technology and Research (A*STAR), 8A Biomedical Grove, Neuros/Immunos #06-04/05, Singapore 138648, Singapore; shiminjiang@gmail.com (S.J.); cjbrown@imcb.a-star.edu.sg (C.J.B.); 5 Department of Chemical and Biomolecular Engineering, Carl R. Woese Institute for Genomic Biology, University of Illinois at Urbana-Champaign, Urbana, IL 61801, USA; 6 Synthetic Biology Translational Research Program, Yong Loo Lin School of Medicine, National University of Singapore, 10 Medical Drive, Singapore 117597, Singapore

**Keywords:** halogenation, heteroaromatic, X-ray crystallography, flavin-dependent halogenases

## Abstract

RadH is one of the flavin-dependent halogenases that has previously exhibited promising catalytic activity towards hydroxycoumarin, hydroxyisoquinoline, and phenolic derivatives. Here, we evaluated new functional homologs of RadH and expanded its specificities for the halogenation of non-tryptophan-derived, heterocyclic scaffolds. Our investigation revealed that RadH could effectively halogenate hydroxyquinoline and hydroxybenzothiophene. Assay optimization studies revealed the need to balance the various co-factor concentrations and where a GDHi co-factor recycling system most significantly improves the conversion and efficiency of the reaction. A crystal structure of RadH was also obtained with a resolution of 2.4 Å, and docking studies were conducted to pinpoint the binding and catalytic sites for substrates.

## 1. Introduction

In nature, there exist thousands of halogenated natural products, with chlorinated ones being the most common, followed by brominated, iodinated, and fluorinated, respectively. Biological investigations revealed that the subtle introduction of halogens in these metabolites often results in unexpected specific and unique bioactivities which are absent in their parent compounds. Consequently, halogenated molecules are important constituents of numerous modern agrochemicals, pharmaceuticals, polymers, and specialty chemicals today [1,2,3]. The industrial halogenation process to obtain key synthetic intermediates that constitute up to 55% of chemical and 85% of pharmaceutical products relies on the chloroalkali electrolysis process, which uses strong acids for pH adjustments, generating polluting chlorinated solvents, and operates in highly hazardous plants that consume a lot of energy. As the chemical industries pivot to more sustainable and energy-conserving processes, there is a strong motivation to identify more environmentally benign methods to halogenate compounds.

Recent advances in enzymatic halogenation have afforded opportunities to circumvent challenges with traditional chemical approaches and opened up possibilities for chemo- and regio-selective C-H halogenation [4]. Flavin-dependent halogenases (FDHs) have been pivotal in this domain, given their ability to site-selectively install halogens across a wide range of electron-rich aromatic structures [5,6]. The development of enzymatic halogenation tools among FDHs has been largely centered on prokaryote-derived tryptophan halogenases, including the elucidation of FDH’s mechanism of action based on structural insights from PrnA, RebH, and PyrH [7,8,9]. An activity profiling study for tryptophan halogenases revealed a substrate scope that consists of indole, pyrrole, and aniline derivatives, which are sparsely utilized by the less explored phenolic halogenases [10]. Instead, a series of hydroxyl-conjugated macrolactones and polycyclic compounds have been found to be accepted by this group of fungal-related enzymes [11,12]. Such in-depth characterization of non-tryptophan halogenases can facilitate access to a wider range of privileged scaffolds, which are often associated with natural products of biological relevance. 

In particular, the fungal halogenase RadH from *Chaetomium chiversii* has been shown to be particularly promising in the tailoring of non-tryptophan-derived scaffolds due to the relatively broad range of reactions that can be catalyzed [13]. Based on the study by Menon et al., RadH has demonstrated distinct catalytic activity towards hydroxycoumarin, hydroxyisoquinoline, and phenolic derivatives, in addition to its native substrate Monocillin II. Given the promiscuous nature of RadH and reports of modest activity beyond phenolic compounds from related enzymes, there are reasons to suggest the substrate scope of this functional halogenase can be broader than expected [10,14]. Herein, we report the characterization of new functional homologs of RadH, along with expanded substrate specificities towards new heterocyclic scaffolds for this class of enzymes and the first crystal structure of RadH. 

## 2. Materials and Methods

### 2.1. Halogenase Cloning, Expression, and Purification

The RadH gene was codon optimized, synthesized (Twist Biosciences, South San Francisco, CA, USA), and cloned into a pET28 vector. The final construct was transformed into T7 Express *Escherichia coli* (New England Biolabs, Ipswich, MA, USA) for expression. The resulting strain was cultured in 1 L LB media at 37 °C. When OD_600_ reached 0.4–0.6, 0.1 mM isopropyl β-D-1-thiogalactopyranoside (IPTG) was used to induce overnight expression at 16 °C. After expression, the cultures were centrifuged at 10,000× *g* for 10 min at 4 °C. The resulting pellets were resuspended in 20 mL of 100 mM sodium phosphate pH 7, 10 mM imidazole, and 150 mM sodium chloride before sonication. After sonication, the resulting lysate was then centrifuged at 19,000× *g* for 1 h at 4 °C. The supernatant was incubated with Ni-NTA agarose for 1 h at 4 °C. The resin was washed with 20 mL of 100 mM sodium phosphate pH 7, 80 mM imidazole, and 150 mM sodium chloride, and the bound protein was eluted with 5 mL of 100 mM sodium phosphate pH 7, 500 mM imidazole, and 50 mM sodium chloride. The elution was buffer exchanged and concentrated with 50 mM sodium phosphate pH 7, 10% glycerol and concentrated with 50 mM sodium phosphate pH 7, 10% glycerol. 

### 2.2. Method for Flavin Reductase (Fre) Cloning, Expression, and Purification 

The nucleic acid sequence that encodes for Flavin reductase Fre was purchased as a gBlock from Integrated DNA Technologies, Singapore. The Fre sequence was cloned into a pET-28a(+) vector via the NEBuilder^®^ HiFi DNA Assembly method and transformed into *E*. *coli* Acella (EdgeBio, San Jose, CA, USA). An E. Coli strain expressing Fre was cultured in 1 L of LB Kan50 media at 37 °C. At OD600 0.4–0.6, 0.1 mM IPTG was used to induce protein expression at 16 °C over 18 h. The cell culture was harvested by centrifugation at 4000 rcf for 10 min at 4 °C. After the media was decanted, the cell pellet was resuspended in 30 mL of 50 mM tris pH 7.4, 300 mM sodium chloride, 10 mM imidazole, and lysed by cell disruption. The cell lysate was centrifuged at 33,600 rcf for 45 min at 4 °C to differentiate supernatant from insoluble debris. Fre proteins from lysate supernatant were purified using immobilized metal affinity chromatography via TALON resins interaction with N-terminus His-tag Fre. After lysate supernatant was applied, 10 mL of 50 mM tris pH 7.4, 300 mM sodium chloride, and 10 mM imidazole were used to wash the resins. Fre proteins were eluted from the resins using 5 mL of 50 mM tris pH 7.4, 300 mM sodium chloride, and 200 mM imidazole. Eluted samples were buffer exchanged and concentrated with 50 mM tris pH 7.4, 100 mM NaCl, and 10% glycerol.

### 2.3. Glucose Dehydrogenase from Pseudomonas sp. (Gdhi)

Purchased from Sigma-Aldrich, Burlington, MA, USA with activity units ≥ 200 U/mg.

### 2.4. Crystallization and X-ray Crystallography of RadH

After Ni-NTA purification, RadH proteins were further purified using fast protein liquid chromatography (FPLC). The elute was diluted two-fold with 100 mM sodium phosphate pH 7 before loading onto a HiTrap-Q anion exchange column for FPLC (AKTA start, GE Healthcare life sciences, buffer A: 100 mM sodium phosphate pH 7, buffer B: 100 mM sodium phosphate pH 7, 1 M sodium chloride). A linear gradient was used to elute the protein. Fractions containing the protein of interest were combined and buffer-exchanged into 50 mM Tris, pH 7 (Figure S1). RadH crystals were grown by vapor diffusion using the hanging–drop method with drops containing 0.5 µL of RadH protein (2–3 mg/mL) and 0.5 µL of the reservoir buffer (0.1 M MES, pH 6.5, 40% PEG200). After the drops were incubated at 18 °C, rod-shaped crystals appeared in 3–4 weeks. The crystals were directly picked from the original drop and then flash-frozen to 100 K with liquid nitrogen. X-ray diffraction data were collected at Australian Synchrotron beamline MX2. Data were processed with XDS [15] and elliptically truncated with STARANISO [16]. To solve the structure, molecular replacement using the structure of halogenase CndH from *Chondromyces crocatus* (PDB code 3E1T) as a search model was performed with PHASER [17]. The initial model was built using the Autobuild of PHENIX [18]. Subsequently, substantial manual building was carried out in the program ISOLDE [19] with iterative rounds of refinement using PHENIX [18]. The structure quality was validated using the MOLPROBITY web server [20]. Molecular structure figures were generated using the program PyMOL (Schrödinger, New York City, NY, USA). Coordinate and structure factor amplitude was deposited in the Protein Data Bank with the submission code 8GU0. Table S1 shows data collection and structure refinement statistics.

### 2.5. Molecular Docking

Docking of 6-hydroxyquinoline was performed on the RadH crystal structure using DiffDock [21], a diffusion-generative model over the non-Euclidean manifold of ligand poses. The RadH.pdb file and SMILES string of 6-hydroxyquinoline were used as inputs. Default parameters (20 inference steps and 40 samples obtained) were applied to the model, which was trained on the standard PDBBind database. This manifold of ligand poses was then denoised via reverse diffusion over translational, rotational, and torsional degrees of freedom and weighted against provided model weights. The models were then ranked according to their confidence levels, with the highest confidence model being used in our analysis.

### 2.6. RadH-Catalyzed Halogenation

In a solution containing the quinolinol derivatized starting material (0.5 mM), MgCl_2_ (10 mM), glucose (5.0 mM), flavin adenine dinucleotide disodium salt hydrate (FAD) (1.0 µM), RadH (12.5 µM), flavin reductase (Fre) (2.5 µM), and glucose dehydrogenase from *Pseudomonas sp.* (Gdhi) (2.5 µM) in 10 mM potassium phosphate buffer, reduced nicotinamide adenine dinucleotide (NADH) (2.5 mM) was added to a total volume of 200 µL. After an overnight incubation of 30 °C and orbital shaking at 350 rpm, reactions were quenched with an equivalent volume of MeOH, pelleted by centrifugation (15,000 rpm for 10 min), and the supernatant was analyzed by HPLC-MS using the analytical HPLC method.

### 2.7. Determination of Halogenation Site

RadH was overexpressed in *E. coli* T7 Express (DE3) cells (NEB). LB medium containing 50 µg/mL kanamycin was used for the preparation of a starter culture grown overnight at 37 °C. The cultures were diluted 1:100 and cultured in LB at 37 °C, 200 rpm until OD_600_ reached 0.4–0.6. Then, 0.1 mM isopropyl β-D-1-thiogalactopyranoside (IPTG) was added to induce the expression of proteins. The pyrrolic derivative starting material (ca. 0.05 mM) was added three hours after IPTG induction, and the cultures were maintained at 25 °C, 200 rpm for another 45 h. Using a centrifuge maintained at 4 °C, the culture was spun down at 10,000× *g* to separately harvest the supernatant and pellet for analysis. The aqueous LB layer was 3× extracted with 300 mL of ethyl acetate while the pellets were vortex, sonicated, and extracted with additional (3 × 50 mL) MeOH. The combined organic layers were filtered through a plug of celite and dried over anhydrous sodium sulphate. The solvent was concentrated in vacuo followed by purification using preparative thin-layer chromatography. The halogenation site was determined by 2D NMR.

### 2.8. Reagents and Materials

All chemicals were purchased from Sigma-Aldrich (USA) Alfa Aesar (Haverhill, MA, USA), Merck (St. Louis, MO, USA), and TCI Global (Singapore) and were used as received. Chemicals and anhydrous solvents were obtained from Sigma-Aldrich and were used without further purification. Spectroscopic grade solvents were purchased from Sigma-Aldrich (USA).

### 2.9. Analytical Methods

Spectroscopic grade solvents were purchased from Sigma-Aldrich. Low-resolution LC-MS spectra were recorded on an Agilent LCMS machine with dual MM-APCI-ES. High-resolution mass spectra (HRMS) were recorded on an Agilent ESI-TOF mass spectrometer at 3500 V emitter voltage. Exact *m*/*z* values are reported in Daltons.

#### 2.9.1. Analytical HPLC Method

In total, 10 µL of the supernatant were injected onto the SecurityGuard™ column (KJ0-4282) with a (4.0 mm × 3.0 mm) guard cartridge before separation using a Phenomenex Gemini^®^ C18 analytical column (5 µm packing, 150 mm × 4.6 mm). Gradient starting conditions of 5% MeCN/H_2_O were held for 1 min before development into 50% MeCN/H_2_O over 3 min, followed by development into 95% MeCN/H_2_O over 3 min. Approximately 95% MeCN/H_2_O was held for 1 min before equilibration returned to starting conditions over 1 min. Starting conditions were held for 1 min, followed by another 2 min post-run. Flow rates were kept constant at 1 mL/ min. The column temperature was kept constant at 30 °C. UV absorbance was detected at 220 nm, 254 nm, and 210 nm throughout the run. 

#### 2.9.2. General LC-MS Method

In total, 10 µL of the supernatant were separated using the appropriate analytical HPLC method described above. Detection was performed using an Agilent^®^ single quadrupole LC/MSD system. 

## 3. Results

### 3.1. New Functional RadH Homologs

To expand on existing enzymatic halogenation toolkit for privileged scaffolds diversification, the discovery of novel protein candidates can be initiated from known enzyme templates [22]. Typically, sequence similarity can be used to relate function, such that protein sequences with significant coherence often imply functional convergence. To survey the sequence space around RadH, a sequence similarity network was generated for the Flavin-dependent halogenase (InterPro family IPR006905) to illustrate the sequence-based relationships of proteins within this functional domain [23]. By applying a 60% identity threshold for network generation, protein sequences were categorized into several putative isofunctional groups, and the cluster associated with RadH was found to contain six other homologous protein sequences, including the known radicicol halogenase, Rdc2 (Figures S2 and S3a). 

**Figure 1:**
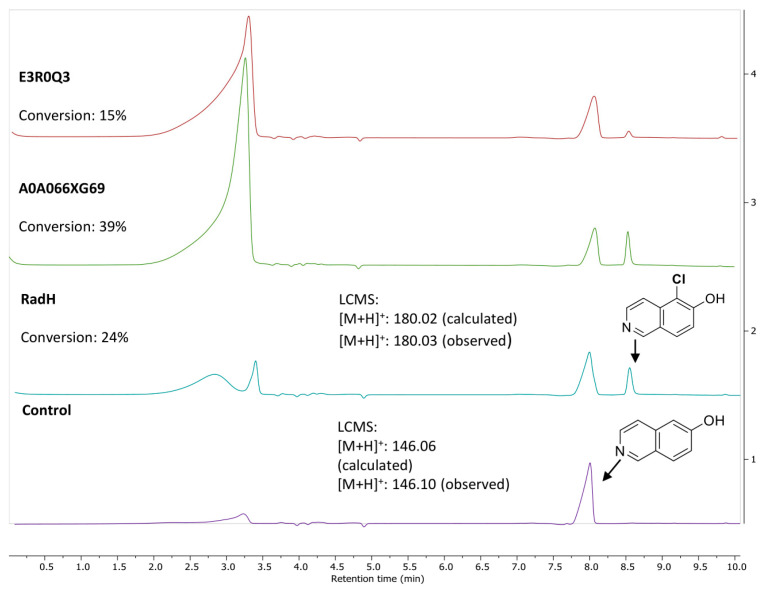
Activity of RadH and the two active homologs against substrate, 6-hydroxyisoquinoline. Conditions: 6-OH isoquinoline (1.0 mM), RadH Biocatalyst (2.0 mol%), FAD (1.0 mol%), Fre (0.4 mol%), NADH (5.0 equiv), MgCl_2_ (10.0 equiv), Tris HCl buffer (10 mM, pH 7.4), 18 h. Control reaction conditions: 6-OH isoquinoline (1.0 mM), FAD (1.0 mol%), Fre (0.4 mol%), NADH (5.0 equiv), MgCl_2_ (10.0 equiv), Tris HCl buffer (10 mM, pH 7.4), and 18 h. Conversion (%) represents the area under the peak (at 254 nm UV absorbance) for the desired chlorinated product (Cl-product) relative to the total area of the Cl-product and remaining starting substrate.

Solubility challenges were observed during heterologous expression of the five uncharacterized fungal protein sequences in *E. coli* (Figure S3b). With the expressed homologs, only two of the five characterized halogenases, namely E3R0Q3 (*Colletotrichum graminicola*) and A0A066XG69 (*Colletotrichum sublineola*), showed activity with standard 6-hydroxyhisoquinoline substrates (Figure 1). Notably, A0A066XG69 demonstrated higher conversion compared to RadH or E3R0Q3 but was expressed with a slightly lower expression yield compared to RadH. As RadH performed most reliably and potentially has a wider scope (*vide infra*), subsequent studies were carried out with RadH. 

### 3.2. Reaction Optimization

Alongside studying substrate specificities, we also hypothesized that enzyme activity could be improved through co-factor recycling and co-factor optimization, building upon our previous research [24]. Thus, the conversion of the standard 6-hydroxyisoquinoline substrate was optimized using RadH (Table 1). Application of the reported conditions using 0.5 mM of 6-hydroxyisoquinoline, RadH (15 µM, 3.0 mol%), along with co-factors FAD (1 µM, 0.2 mol%), Fre (2.5 µM, 0.5 mol%), and NADH (2.5 mM, 5.0 equiv), and chlorinating source, MgCl_2_ (10 mM, 20 equiv), only resulted in 32% conversion after 2 h (Entry 1). Increasing the RadH and co-factor enzyme Fre concentration by ~1.6-fold to 5 mol% and 0.8 mol%, respectively, and increasing the reaction time to 18 h did not increase the conversion (Entry 2). Interestingly, introducing the GdHi recycling system to the assay conditions resulted in a 2.2-fold increase in conversion to 73% (Entry 3). Further finetuning of the co-factors ratio, particularly FAD and NADH concentrations with respect to the GdHi system, did not further improve the conversions (Entry 4), suggesting a fine balance of co-factors ratios is desired for optimum conversions similar to our previous observations [24].

### 3.3. Screen of Substrate Specificities of RadH and Its Homologs

**Figure 2:**
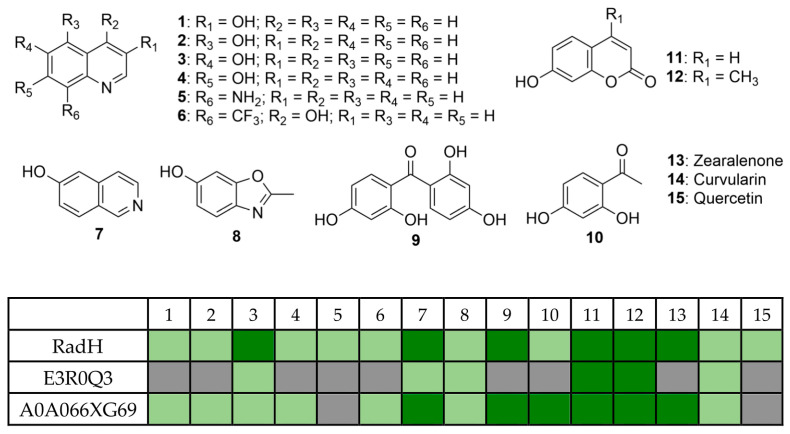
Substrate specificities profile of RadH and the two homologs against a panel of 15 substrates under the halogenation assay. Conditions: substrate (0.5 mM), RadH (3.0 mol%), FAD (0.2 mol%), Fre (0.5 mol%), NADH (5.0 equiv), Glucose (10.0 equiv), GdHi (0.5 mol%), MgCl_2_ (20.0 equiv), and phosphate buffer (10 mM, pH 7.4). Estimated relative % conversions (based on area under the LCMS peaks at 254 nm of starting material and respective product) were color-coded: light green (<5%) and dark green (x ≥ 5%). Grey boxes = not detected by mass spectroscopy.

The catalytic profile of RadH towards hydroxyisoquinoline derivatives was described in an earlier study [13], while the study of RadH acceptance of hydroxyquinolines, a privileged scaffold found in many natural products and drugs, or other less common heterocycles has remained limited. To probe the specificity of RadH towards the lesser-studied structural isomers or uncommon scaffolds, we screened a panel of substrates with different complexity and scaffolds for activity against RadH and its two homologs (Figure 2). Using the above-optimized conditions, RadH showed the widest substrate specificity compared to the other two homologs.

### 3.4. Quinoline and Heterocyclic Substrate Specificities

**Figure 3:**
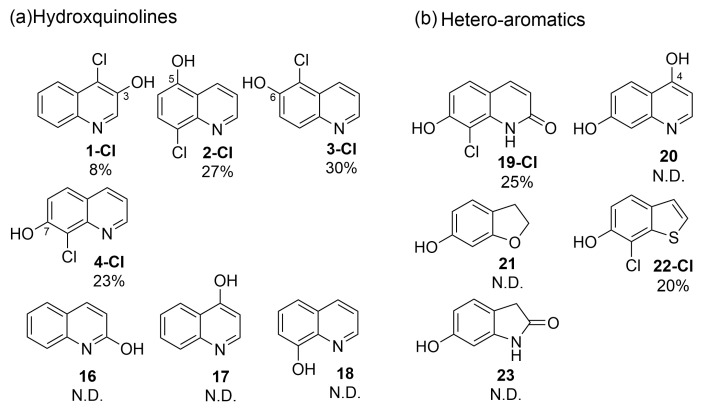
Specificities of RadH. Conditions: Substrate (0.5 mM), RadH Biocatalyst (3 mol%), GdHi (0.5 mol%), FAD (0.2 mol%), Fre (0.5 mol%), NADH (5.0 equiv), Glucose (10.0 equiv), MgCl_2_ (20.0 equiv), and phosphate buffer (10 mM, pH 7.4). Conversions are determined as an average of at least two runs by comparison of the LC peak areas between crude reaction mixtures relative to the control standard. A standard control experiment refers to the reaction setup without the addition of RadH. N.D. = not detected.

To further verify the initial screening, we elected to study the regio-preference of RadH towards hydroxyquinolines in depth (Figure 3a) (See Supporting Information for assignment of the chlorination site by 2D-NMR). RadH was found to accept 3-, 5-, 6-, 7-hydroxyquinolines but not 2-, 4-, 8-hydroxyquinolines, suggesting a slight preference for hydroxyquinolines with OH-moiety attached to the benzene side of the compound. Comparatively, conversion for **1** was much lower compared to substrates **2**, **3**, or **4**, suggesting that deprotonation of a hydroxyl group on the pyridine ring could be more challenging. Our results are in agreement with previous reports by Micklefield and co-workers [13] that **3** was modestly accepted under their conditions but not **18**. 

Compounds with bicyclic 6,5-fused ring systems often constitute the building blocks of bioactive natural products and pharmaceuticals [19]. Therefore, a diverse set of hydroxy-substituted hetero-aromatic substrates was also assessed in order to explore and elucidate the specificities of RadH (Figure 3b). Amongst the five tested substrates of dihydroxyquinolines **19** and **20**, hydroxybenzofuranol **21**, hydroxybenzothiophene **22**, and hydroxyoxindole **23**, only 2,7-dihydroxyquinoline (**19**) and 7-hydroxybenzothiophene (**22**) were found to be accepted modestly by RadH under the optimized conditions. It appears that only substrates with unsaturated heterocycles were tolerated by RadH, suggesting that a bicyclic aromatic structure could be important for substrate binding and recognition. In all cases, halogenation generally occurred ortho to the hydroxyl group, which is consistent with the previous regioselective characterization of RadH. 

### 3.5. Crystal Structure of RadH

**Figure 4:**
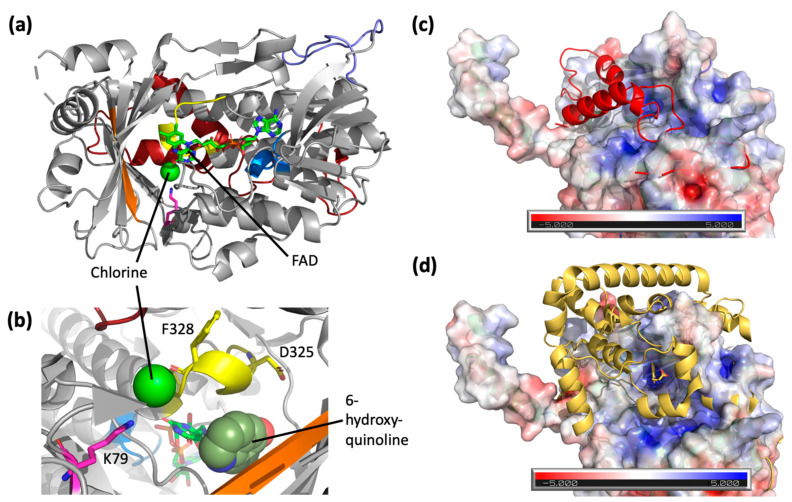
(**a**) Crystal structure of RadH (PDB: 8GU0) with FAD and chlorine atom bound. Main structural features are colored accordingly: GxGxxG domain (blue), Fx.Px.Sx.G domain (yellow), WxWxIP domain (orange), and catalytic lysine residue [8,26] (pink). The additional loop region of RadH (purple) and C-terminus region (red) of RadH are structurally different from other halogenases. (**b**) A zoom-in view of active site residues of RadH. The highest confidence docking pose of 6-hydroxyquinoline in the active site of RadH shown as spheres. (**c**) Overlay of the C-terminus of RadH (red) over the electrostatic potential map of CndH (PDB: 3E1T). (**d**) Overlay of the C-terminus of PrnA (PDB: 2AR8) (yellow) over the electrostatic potential map of CndH. The tyrosine substrate bound to PrnA is sitting at the proposed active site of CndH.

To date, a handful of crystal structures have been resolved for FDHs (Table 2), and there is at least one resolved crystal structure for the major sub-families (Figure S2) [25]. However, there is yet a crystal structure for the sub-family belonging to Rdc2 or RadH. Here, a 2.4 Å resolution crystal structure of RadH was obtained using an analogous FDH as a search model, and the crystalline form of this enzyme revealed two largely identical monomers in an asymmetric unit. Each monomer consists of a conserved Flavin-binding domain with FAD bound and a chlorine atom located in an adjacent space (Figure 4a). RadH contains several structural features thought to contribute towards the halogenase activity of the enzyme, namely the GxGxxG FAD-binding domain, Fx.Px.Sx.G domain spanning the active site and FAD-binding domain, and a WxWxIP domain on the β-sheet bordering the active site, corroborating similar observations of other FDHs structure (Table 2). Docking of 6-hydroxyquinoline was performed using DiffDock [21] and suggested that the active site of RadH be the space currently occupied by the flavin moiety of FAD and chlorine atom (Figure 4b). While the docked poses overlap with the FAD structure, previous experimental results [13] have shown that nearby residues K74, D325, and F328 are essential for RadH activity, validating the RadH active site proposed by the docking model.

A comparison of RadH to structural homologues uncovered two new features unique to RadH. The closest structural homolog of RadH is the FAD-dependent chondrochloren halogenase CndH with the lowest RMSD score of 2.39 and 29% sequence identity (Table 2). Spatial superimposition has revealed highly similar folds between the two proteins, except for an additional omega loop (Y138–P154) in RadH (Figure 4a). This appears to be unique to RadH when compared to other known FDHs (Figures S3–S6). CndH is a variant of B FDH, catalyzing reactions only on substrates attached to carrier proteins [27,28]. In contrast, variant A FDHs [29,30,31] are able to act on free substrates. The binding of carrier proteins is suggested to occur at a hydrophobic groove on CndH next to the entry of the active site [27]. This groove is also where the C-terminus domain of RadH is located (Figure 4b, top). As a result, we hypothesize that the C-terminus of RadH has a similar structural role as a docked carrier protein, positioning the enzyme in the correct orientation for catalysis. This is similarly seen in other variant A FDHs, such as PrnA [32,33], which has a C-terminus helical lid covering the active site of CndH (Figure 4c, bottom). However, the C-terminus of RadH does not form the full helical lid found in the other variant, A FDHs.

The addition of RadH structural data thereby supplements existing spatial understanding of variant A non-tryptophan FDHs. While RadH is a variant A FDH itself, it has higher structural homology than variant B FDHs [27,28] (Table 2). This implies that while RadH might perform halogenation through a similar mode of action to variant B FDHs, it is likely to have a broader substrate scope associated with variant A FDHs [25], as aligned with our experimental observations.

## 4. Discussion

We evaluated several homologs of the Flavin-dependent enzyme for diversifying privileged scaffolds via enzymatic halogenation based on a sequence similarity network generated from the native enzyme, RadH. Two functional homologs were identified. RadH was found to also accept certain hydroxyquinoline and hydoxybenzothiophene as viable substrates through scaffold screening. This slightly expanded substrate specificity towards [6.5]-fused heteroaromatic substrates is interesting as access to modifications of such scaffolds is important for medicinal chemistry libraries [34,35] or as potential probes for cancer therapy [36]. Our assay optimization revealed the intricacy of balancing various co-factor concentrations, where we showed increased halogenation yields by >2-fold through GDHi co-factor recycling. To improve the activity of the enzyme for practical biocatalytic applications, further engineering of the enzyme is needed, such as the development of a better intermediate transfer tunnel (preventing leakage of hypohalous acid) [37].

In this study, we also present the first crystal structure of RadH. However, despite our efforts, we were unable to obtain structures of substrate-bound RadH. Nonetheless, we were able to identify putative catalytic sites within RadH by computational docking. Further studies are required to investigate the precise location and binding of substrates, as well as to identify the residues responsible for substrate specificities of RadH. These studies are crucial for expanding the promiscuity of RadH and enabling its application in a wider range of fields.

## Figures and Tables

**Table 1:**
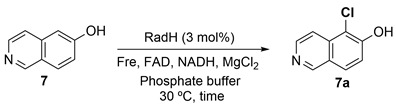
Optimization of assay conditions of RadH against 6-hydroxyisoquinoline.

Entry	Variation from Standard Conditions ^a^	Time (h)	Conversion (%) ^b^
1	None	2	32
2	5 mol% RadH, 0.8 mol% of Fre	18	30
3	Addition of 0.5 mol% GdHi	18	73
4	20 mol% of FAD, 25 µM of NADH	18	31

a, standard Conditions: 6-OH isoquinoline (0.5 mM), RadH (3.0 mol%), FAD (0.2 mol%), Fre (0.5 mol%), NADH (5.0 equiv), Glucose (10.0 equiv), MgCl_2_ (20.0 equiv), and phosphate buffer (10 mM, pH 7.4). b, conversion (%) represents the area under the peak (at 254 nm UV absorbance) for the desired chlorinated product (Cl-product) relative to the total area of the Cl-product and remaining starting substrate. Note: For each of the entries, a control experiment without the addition of RadH was also carried out.

**Table 2:** Alignment data of RadH against selected halogenases with known structures arranged in decreasing root mean square deviation (RMSD). The template modeling score (TM-score) and sequence identity percentage (Sequence ID) are also listed.

Halogenase	Variant	RMSD	TM-Score	Sequence ID (%)
CndH	B	2.39	0.81	29
PltA	B	2.65	0.84	26
Mpy16	B	2.7	0.84	27
CMIS	A	2.83	0.66	26
PltM	A	3.3	0.7	18
PyrH	A	4.05	0.63	17
PrnA	A	4.08	0.62	17
RebH	A	4.14	0.61	19

## Data Availability

Data supporting the results are shown in the paper and are available upon reasonable request.

## Supplementary Materials

The following supporting information can be downloaded at: https://www.mdpi.com/2218-273X/13/7/1081/s1, Figure S1: Ni-NTA purification; Figure S2: There are several putative isofunctional protein sequence clusters which are associated with RadH, and one cluster contains the known radicicol halogenase, Rdc2; Figure S3: Sequence similarity network for Flavin-dependent halogenase InterPro family IPR006905, cluster containing known halogenase sequence labelled with corresponding color. (A) Sequence alignment of RadH and homologs, putative active site amino acid residues highlighted in yellow and conserved WxWxIP motif in red box. (B) Sequence identity of homologs to RadH and protein yields in *E. coli*; Figure S4: Structural alignment of RadH with CndH. RadH and CndH denoted in orange and blue, respectively. Structural features of interest for RadH and CndH are shown in cream and grey, respectively; Figure S5: Structural alignment of RadH with PltM. RadH and PltM denoted in orange and blue, respectively. Structural features of interest for RadH and PltM are shown in cream and grey, rexpectively; Figure S6: Structural alignment of RadH with PrnA. RadH and PrnA denoted in orange and blue, respectively. Structural features of interest for RadH and PrnA are shown in cream and grey, rexpectively; Figure S7: Structural alignment of RadH with RebH. RadH and RebH denoted in orange and blue, respectively. Structural features of interest for RadH and RebH are shown in cream and grey, respectively; Figure S8: LCMS chromatogram for enzymatic reaction and its control for product 1-Cl; Figure S9: LCMS chromatogram for enzymatic reaction and its control for product 2-Cl; Figure S10: LCMS chromatogram for enzymatic reaction and its control for product 3-Cl; Figure S11: LCMS chromatogram for enzymatic reaction and its control for product 4-Cl; Figure S12: LCMS chromatogram for enzymatic reaction and its control for product 19-Cl; Figure S13: LCMS chromatogram for enzymatic reaction and its control for product 22-Cl; Figure S14: ^1^H and ^13^C NMR spectrum for 1-Cl; Figure S15: ^1^H-^1^H COSY and ^1^H-^13^C HSQC spectrum for 1-Cl; Figure S16: ^1^H-^13^C HMBC and ^13^C DEPT135 spectrum for 1-Cl; Figure S17: ^1^H and ^13^C NMR spectrum for 2-Cl; Figure S18: ^1^H-^1^H COSY and ^1^H-^13^C HSQC spectrum for 2-Cl; Figure S19: ^1^H-^13^C HMBC spectrum for 2-Cl; Figure S20: ^1^H and ^13^C NMR spectrum for 3-Cl; Figure S21: ^1^H-^1^H COSY and ^1^H-^13^C HMBC spectrum for 3-Cl; Figure S22: ^1^H-^13^C HSQC spectrum for 3-Cl; Figure S23: ^1^H and ^13^C NMR spectrum for 4-Cl; Figure S24: ^1^H-^1^H COSY and ^1^H-^13^C HMBC spectrum for 4-Cl; Figure S25: ^1^H-^13^C HSQC spectrum for 4-Cl; Figure S26: ^1^H and ^13^C NMR spectrum for 19-Cl; Figure S27: ^1^H-^1^H COSY and ^1^H-^13^C HSQC spectrum for 19-Cl; Figure S28: ^1^H-^13^C HMBC spectrum for 19-Cl; Figure S29: ^1^H and ^13^C NMR spectrum for 22-Cl; Figure S30: ^1^H-^1^H COSY and ^1^H-^13^C HSQC spectrum for 22-Cl; Figure S31: ^1^H-^13^C HMBC spectrum for 22-Cl.; Table S1: Data collection and structural refinement for RadH crystal.
