# Supplementary material for: Further Characterization of Fungal Halogenase RadH and Its Homologs
Source: Biomolecules. 2023 Jul 6;13(7):1081. doi: 10.3390/biom13071081 (PMC10377541; doi:10.3390/biom13071081)
Supplement: Supplementary file 1 [file biomolecules-13-01081-s001.zip › biomolecules-2451820-supplementary.pdf]

Supporting Information

Further Characterization of the fungal halogenase RadH and its homologs

GuangRong Peh [1], Gregory A. Gunawan [1,2], Terence Tay [3], Elaine Tiong [2], Lee Ling Tan [2], Shimin Jiang [4], Yi Ling Goh [1], Suming Ye [1], Joel Wong [1], Chris Brown [4], Huimin Zhao [3,5], Ee Lui Ang [3,6,*], Fong Tian Wong [1,2,*] and Yee Hwee Lim [1,6,*]

1 Institute of Sustainability for Chemicals, Energy and Environment (ISCE²), Agency for Science, Technology and Research (A*STAR), 8 Biomedical Grove, Neuros #07-01, Singapore 138665, Republic of Singapore.

2 Institute of Molecular and Cell Biology (IMCB), Agency for Science, Technology and Research (A*STAR), 61 Biopolis Dr, Proteos #07-01, Singapore 138673, Republic of Singapore.

3 Singapore Institute of Food and Biotechnology Innovation (SIFBI), Agency for Science, Technology and Re-search (A*STAR), 31 Biopolis Way, Nanos #01-02, Singapore 138669, Republic of Singapore.

4 Disease Intervention Technology Laboratory, Institute of Molecular and Cellular Biology, Agency for Science, Technology and Research (A*STAR), 8A Biomedical Grove, #06 04/05 Neuros/Immunos, Singapore 138648, Singapore.

5 Department of Chemical and Biomolecular Engineering, Carl R. Woese Institute for Genomic Biology. University of Illinois at Urbana-Champaign, Urbana, IL, USA.

6 Synthetic Biology Translational Research Program, Yong Loo Lin School of Medicine, National University of Singapore, 10 Medical Drive, Singapore 117597, Republic of Singapore

*** Correspondence:**

Y.H.L. lim_yee_hwee@isce2.a-star.edu.sg**,**

F.T.W. wongft@imcb.a-star.edu.sg**,**

E.L.A. ang_ee_lui@sifbi.a-star.edu.sg

Contents

1. Reagents and materials 4

2. Supplementary Tables and Figures 6

a) Data collection and structural refinement for RadH crystal. 6

b) Two-step purification of RadH. 7

Figure S1: Ni-NTA purification 7

c) Generating a Sequence Similarity Network for the Flavin-dependent Halogenase InterPro Family IPR006905 7

Figure S2: There are several putative isofunctional protein sequence clusters which are associated with RadH, and one cluster contains the known radicicol halogenase, Rdc2. 7

Figure S3: Sequence similarity network for Flavin-dependent halogenase InterPro family IPR006905 8

c) Spatial superimposition of RadH to structural homologues 9

Figure S4. Structural alignment of RadH with CndH 9

Figure S5. Structural alignment of RadH with PltM 9

Figure S6. Structural alignment of RadH with PrnA 10

Figure S7. Structural alignment of RadH with RebH 10

d) LC-MS analytical data for substrates panel using purified RadH 11

Figure S8. LCMS chromatogram for enzymatic reaction and its control for product 1-Cl. 11

Figure S9. LCMS chromatogram for enzymatic reaction and its control for product 2-Cl. 12

Figure S10. LCMS chromatogram for enzymatic reaction and its control for product 3-Cl. 13

Figure S11. LCMS chromatogram for enzymatic reaction and its control for product 4-Cl. 14

Figure S12. LCMS chromatogram for enzymatic reaction and its control for product 19-Cl. 15

Figure S13. LCMS chromatogram for enzymatic reaction and its control for product 22-Cl 16

c) NMR Spectra of selected halogenated products 17

Figure S14. ^{1}H and ^{13}C NMR spectrum for 1-Cl. 17

Figure S15. ^{1}H-^{1}H COSY and ^{1}H-^{13}C HSQC spectrum for 1-Cl. 18

Figure S16. ^{1}H-^{13}C HMBC and ^{13}C DEPT135 spectrum for 1-Cl. 19

Figure S17. ^{1}H and ^{13}C NMR spectrum for 2-Cl. 20

Figure S18. ^{1}H-^{1}H COSY and ^{1}H-^{13}C HSQC spectrum for 2-Cl. 21

Figure S19. ^{1}H-^{13}C HMBC spectrum for 2-Cl. 22

Figure S20. ^{1}H and ^{13}C NMR spectrum for 3-Cl. 23

Figure S21. ^{1}H-^{1}H COSY and ^{1}H-^{13}C HMBC spectrum for 3-Cl 24

Figure S22. ^{1}H-^{13}C HSQC spectrum for 3-Cl. 25

Figure S23. ^{1}H and ^{13}C NMR spectrum for 4-Cl. 26

Figure S24. ^{1}H-^{1}H COSY and ^{1}H-^{13}C HMBC spectrum for 4-Cl 27

Figure S25. ^{1}H-^{13}C HSQC spectrum for 4-Cl. 28

Figure S26. ^{1}H and ^{13}C NMR spectrum for 19-Cl. 29

Figure S27. ^{1}H-^{1}H COSY and ^{1}H-^{13}C HSQC spectrum for 19-Cl. 30

Figure S28. ^{1}H-^{13}C HMBC spectrum for 19-Cl. 31

Figure S29. ^{1}H and ^{13}C NMR spectrum for 22-Cl. 32

Figure S30. ^{1}H-^{1}H COSY and ^{1}H-^{13}C HSQC spectrum for 22-Cl. 33
Figure S31. ^{1}H-^{13}C HMBC spectrum for 22-Cl. 34

1. Reagents and materials

All chemicals were purchased from Sigma-Aldrich, Alfa Aesar, Merck and TCI and were used as received. Chemicals and anhydrous solvents were obtained from Sigma Aldrich and were used without further purification. Spectroscopic grade solvents were purchased from Sigma Aldrich. Proton (^{1}H NMR) and carbon (^{13}C NMR) nuclear magnetic resonance spectra were recorded on a Bruker Avance 400 spectrometer with CryoProbe at 400 MHz and 100 MHz. The chemical shifts are reported in parts per million (ppm) on the delta (δ) scale. The solvent peak was used as a reference value, for ^{1} H NMR: $CDCl_3$ = 7.27 ppm, CD_3OD = 3.31 ppm, CD_2Cl_2 = 5.32 ppm for ^{13}C NMR: $CDCl_3$ = 77.23, CD_3OD = 49.0 ppm, CD_2Cl_2 = 53.50 ppm. NMR spectra were processed using MestReNova 10.0.2. Data are reported as follows: (s = singlet; d = doublet; t = triplet; q = quartet; sept = septet; dd = doublet of doublets; ddd = doublet of doublet of doublets; dddd = doublet of doublet of doublet of doublet; td = triplet of doublets; dtd = doublet of triplet of doublets; br = broad). Analytical TLC was performed on E. Merck pre-coated (25 mm) silica gel 60F-254 plates. Visualization was done under UV (254 nm). Flash column chromatography was carried out using Merck 60 F254, 0.040-0.063 μm silica gel. Preparative TLC chromatography was carried out using Merck 60 F254, (250 μm layer thickness) silica gel plates. High-resolution mass spectra (HRMS) were recorded on an Agilent ESI-TOF mass spectrometer at 3500 V emitter voltage.

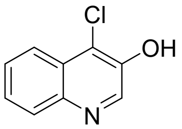

4-chloroquinolin-3-ol (1-Cl): ^{1}H NMR (400 MHz, Methanol-*d*$_4$) δ 8.43 (s, 1H), 7.98 (ddd, *J* = 8.5, 1.4, 0.6 Hz, 1H), 7.81 (dt, *J* = 8.3, 0.9 Hz, 1H), 7.47 (ddd, *J* = 8.4, 6.8, 1.3 Hz, 1H), 7.33 (ddd, *J* = 8.3, 6.8, 1.4 Hz, 1H). ^{13}C NMR (101 MHz, Methanol-*D*$_4$) δ 159.1, 150.3, 140.9, 130.6, 128.9, 128.0, 124.5, 123.3, 123.2. HRMS (ESI) calcd. for C_9H_7ClNO m/z $(M+H)^+$: 180.0211, found: 180.0253.

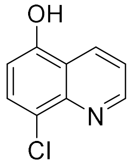

8-chloroquinolin-5-ol (2-Cl): ^{1}H NMR (400 MHz, Methanol-d4) δ 8.81 (ddd, J = 8.4, 1.8, 0.8 Hz, 1H), 8.60 (dd, J = 4.3, 1.8 Hz, 1H), 7.55 (d, J = 8.8 Hz, 1H), 7.29 (dd, J = 8.4, 4.3 Hz, 1H), 7.00 (dd, J = 8.9, 0.8 Hz, 1H). ^{13}C NMR (101 MHz, Methanol-*D*$_4$) δ 162.4, 150.0, 149.6, 135.7, 133.5, 127.1, 119.1, 117.3, 112.0. HRMS (ESI) calcd. for C_9H_7ClNO m/z $(M+H)^+$: 180.0211, found: 180.0203.

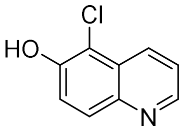

5-chloroquinolin-6-ol (3-Cl): 1H NMR (400 MHz, Methanol-d4) δ 8.74 (dd, J = 4.3, 1.6 Hz, 1H), 8.61 (ddd, J = 8.6, 1.6, 0.8 Hz, 1H), 7.91 (dd, J = 9.2, 0.8 Hz, 1H), 7.61 (dd, J = 8.6, 4.3 Hz, 1H), 7.52 (d, J = 9.2 Hz, 1H). ^{13}C NMR (101 MHz, Methanol-*D*$_4$) δ 153.7, 148.7, 144.6, 133.4, 129.4, 129.3, 123.7, 123.6, 114.0. HRMS (ESI) calcd. for C_9H_7ClNO m/z $(M+H)^+$: 180.0211, found: 180.0217.

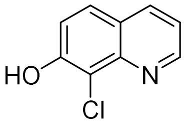

8-chloroquinolin-7-ol (4-Cl): ^{1}H NMR (400 MHz, Methanol-d_4) δ 8.61 (dd, *J* = 4.5, 1.7 Hz, 1H), 8.06 (dd, *J* = 8.0, 1.7 Hz, 1H), 7.51 (d, *J* = 9.0 Hz, 1H), 7.16 (d, *J* = 9.0 Hz, 1H), 7.09 (dd, *J* = 8.0, 4.5 Hz, 1H). ^{13}C NMR (101 MHz, Methanol-D_4) δ 167.6, 150.1, 148.3, 137.9, 127.6, 127.2, 123.1, 116.4, 115.5. HRMS (ESI) calcd. for C_9H_7ClNO m/z $(M+H)^+$: 180.0211, found: 180.0265.

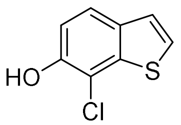

7-chlorobenzo[b]thiophen-6-ol (22-Cl): ^{1}H NMR (400 MHz, Methanol-d_4) δ 7.63 (d, *J* = 8.5 Hz, 1H), 7.40 (d, *J* = 5.4 Hz, 1H), 7.32 (d, *J* = 5.5 Hz, 1H), 7.05 (d, *J* = 8.6 Hz, 1H). ^{13}C NMR (101 MHz, Methanol-D_4) δ 151.8, 151.8, 142.2, 135.6, 125.7, 125.5, 123.7, 116.6, 114.0. HRMS (ESI) calcd. for C_8H_4ClOS m/z $(M-H)^-$: 182.9677, found: 182.9682.

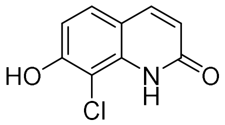

8-chloro-7-hydroxyquinolin-2(1H)-one (19-Cl): ^{1}H NMR (400 MHz, Methanol-d_4) δ 8.55 (s, 1H), 7.86 (d, *J* = 9.4 Hz, 1H), 7.46 (d, *J* = 8.7 Hz, 1H), 6.87 (d, *J* = 8.7 Hz, 1H), 6.41 (d, *J* = 9.4 Hz, 1H). ^{13}C NMR (101 MHz, Methanol-D_4) δ 165.5, 158.5, 143.0, 137.8, 128.7, 117.7, 115.1, 114.1, 106.2. LCMS (Dual MM APCI-ESI) calcd. for $C_9H_7ClNO_2$ m/z $(M+H)^+$: 196.02, found: 194.04.

2. Supplementary Tables and Figures

a) Data collection and structural refinement for RadH crystal.

Table S1.

Data collection	
Resolution (Å)	49.10-2.42 (2.60-2.42)
Space Group	P4
Unit Cell Dimensions (Å)	a = b = 135.17, c = 52.69, α = β = γ = 90°
Temp (K)	100
Multiplicity	6.8 (6.6)[a]
No of Unique Reflections	22240(1112)
Completeness (%)	91.3(62.0)
Rmerge	0.111 (1.398)
I/sigma	11.1 (1.3)
Structure refinement statistics	
R factor (%)	22.85
R free (%)	27.69
RMS Bonds (Å)	0.002
RMS Angles (°)	0.573
No of atoms	
Protein	6933
water	187
Other	198
Average B-Factors (Å)	
Protein	67.33
Water	61.31
Other	66.72
Ramachandran plot (%)	
Favored Region	95.5
Allowed Region	4.5
Outlier Region	0
Ligands/Ions	FAD (2)
	Chloride (1)
PDB code	8GU0

[a]Values for the highest resolution shell are stated in parentheses

b) Two-step purification of RadH.

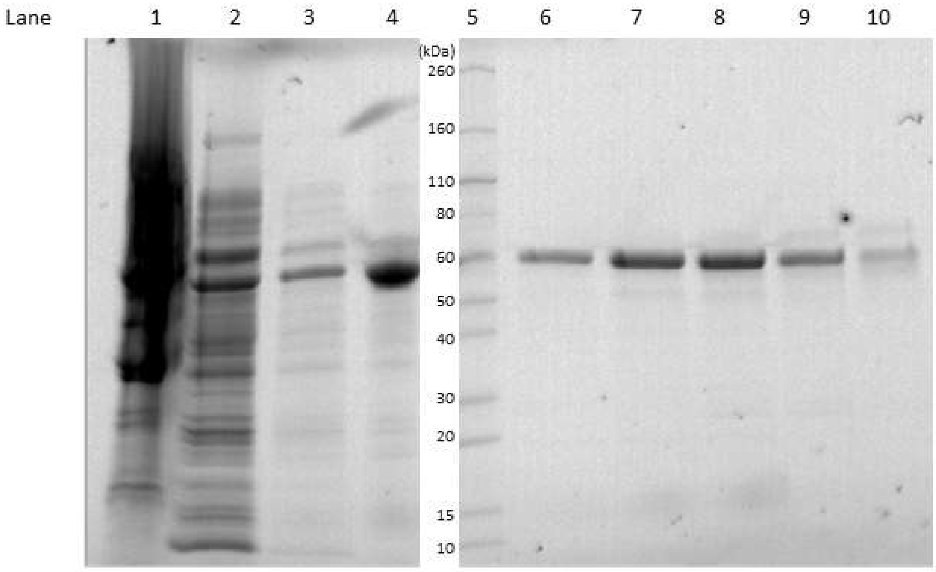

Figure S1. Ni-NTA purification

1st step: A sample of the whole cell lysate (lane 1), the flow-through fraction (lane 2), the wash fraction (lane 3), and the elution fraction (lane 4). FPLC purification 2nd step: A sample which correspond to a peak on the fast phase liquid chromatography was taken for the validation (lanes 6-10). Novex Sharp Unstained protein standards was used for protein size determination (lane 5). RadH has a molecular weight of 56.46 kDa.

c) Generating a Sequence Similarity Network for the Flavin-dependent Halogenase In-terPro Family IPR006905

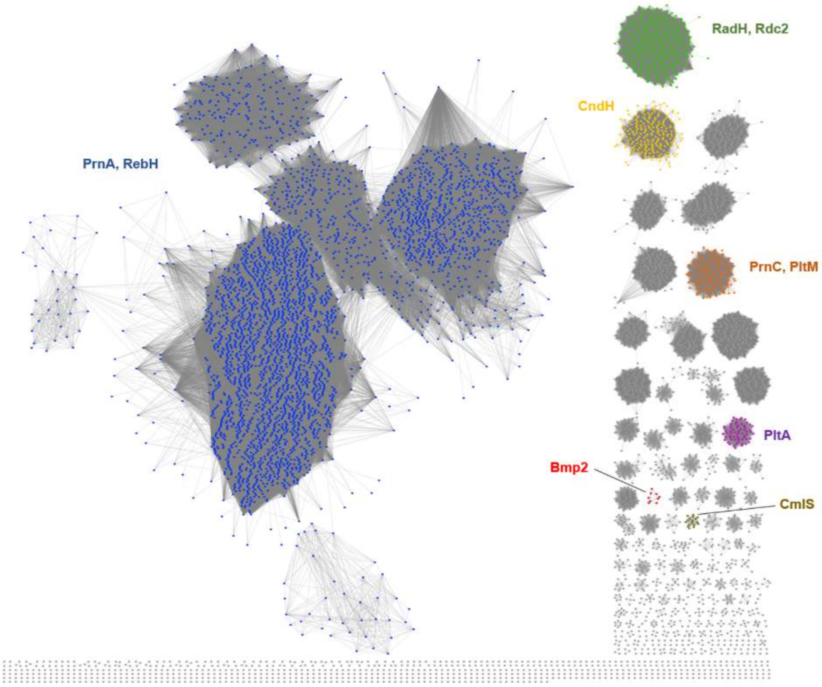

Figure S2. There are several putative isofunctional protein sequence clusters which are associated with RadH, and one cluster contains the known radicicol halogenase, Rdc2.

a)

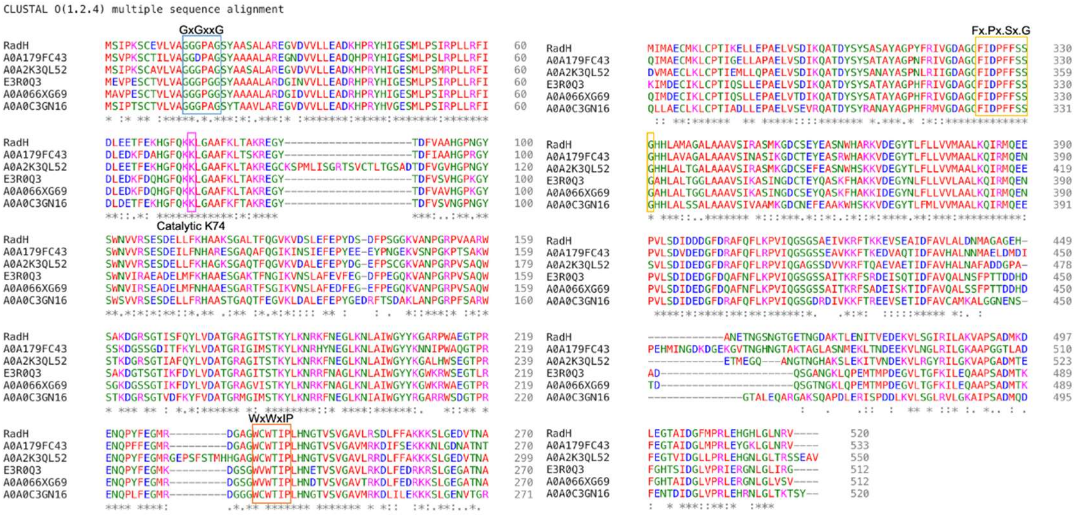

b)

Protein	Sequence identity (%) to RadH	Yield (mg/L)
RadH	100	12
A0A179FC43	75.2	0.56
A0A2K3QL52	79.2	0.46
E3R0Q3	72.8	6.4
A0A066XG69	73.4	9.6
A0A0C3GN16	74.3	1.25
Rdc2	73.5	NA

Figure S3. Sequence similarity network for Flavin-dependent halogenase InterPro family IPR006905, cluster containing known halogenase sequence labelled with corresponding color. A) Sequence alignment of RadH and homologs, putative active site amino acid residues highlighted in yellow and conserved WxWxIP motif in red box. B) Sequence identity of homologs to RadH and protein yields in E.coli.

c) Spatial superimposition of RadH to structural homologues

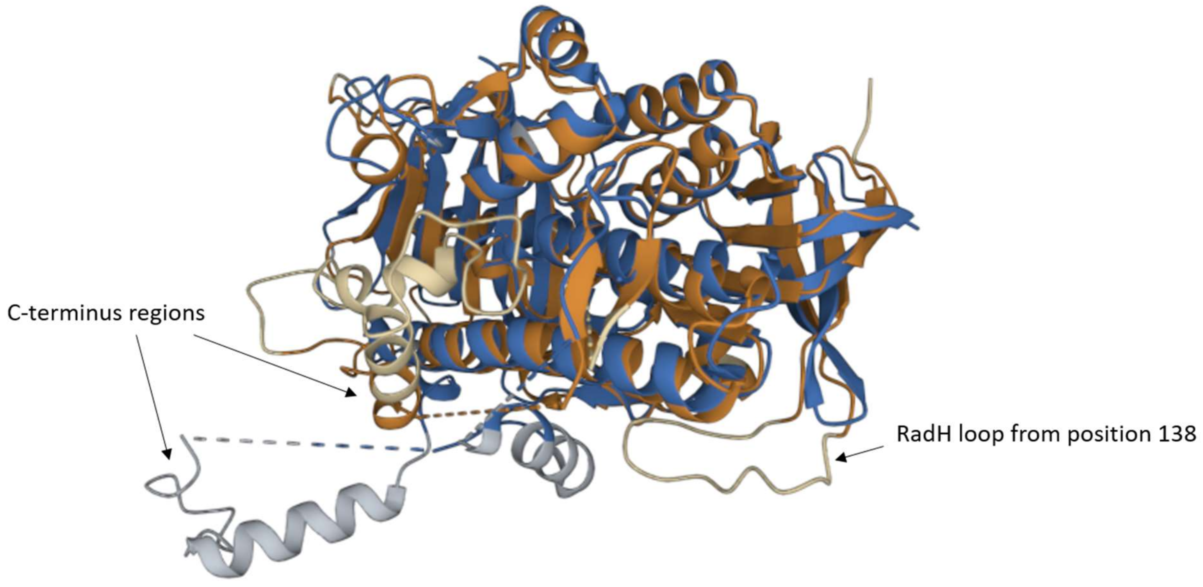

Figure S4. Structural alignment of RadH with CndH. RadH and CndH denoted in orange and blue, respectively. Structural features of interest for RadH and CndH are shown in cream and grey, respectively.

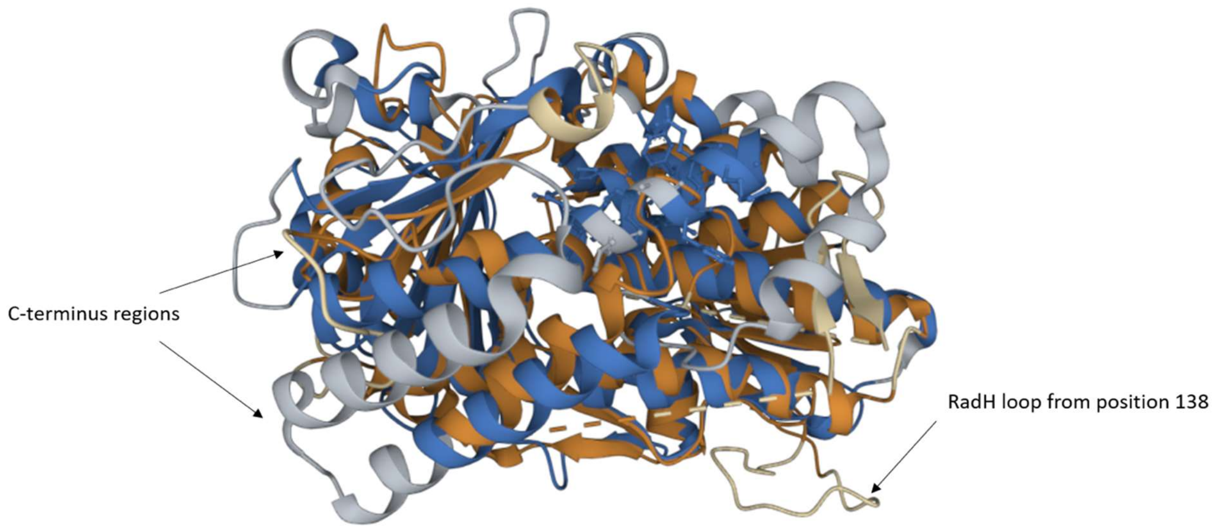

Figure S5. Structural alignment of RadH with PltM. RadH and PltM denoted in orange and blue, respectively. Structural features of interest for RadH and PltM are shown in cream and grey, rexpectively.

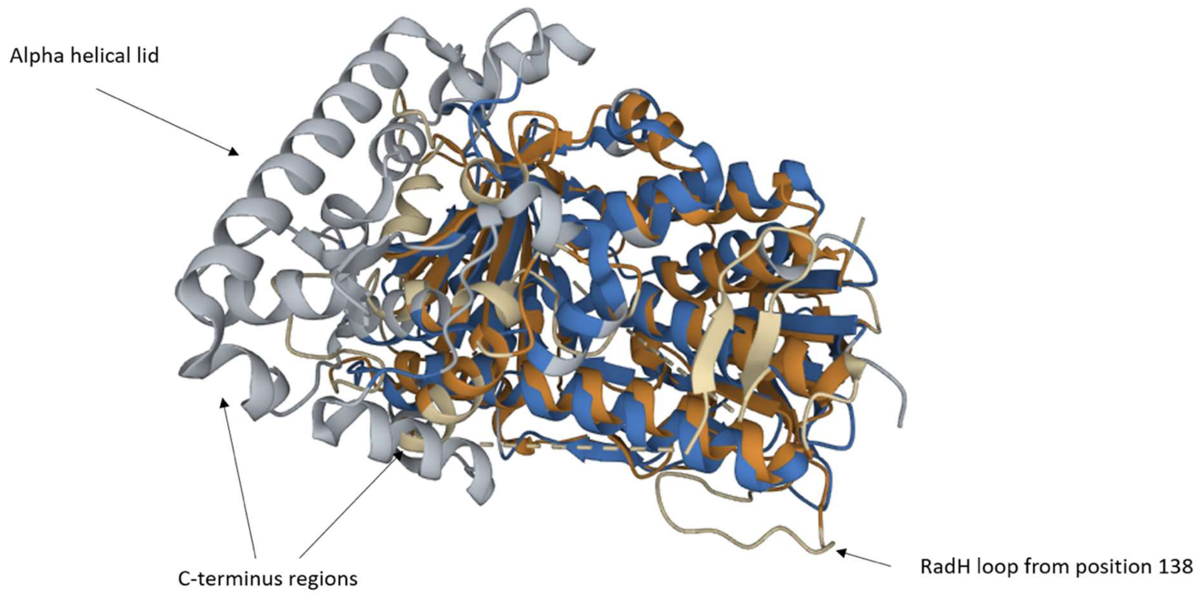

Figure S6. Structural alignment of RadH with PrnA. RadH and PrnA denoted in orange and blue, respectively. Structural features of interest for RadH and PrnA are shown in cream and grey, rexpectively.

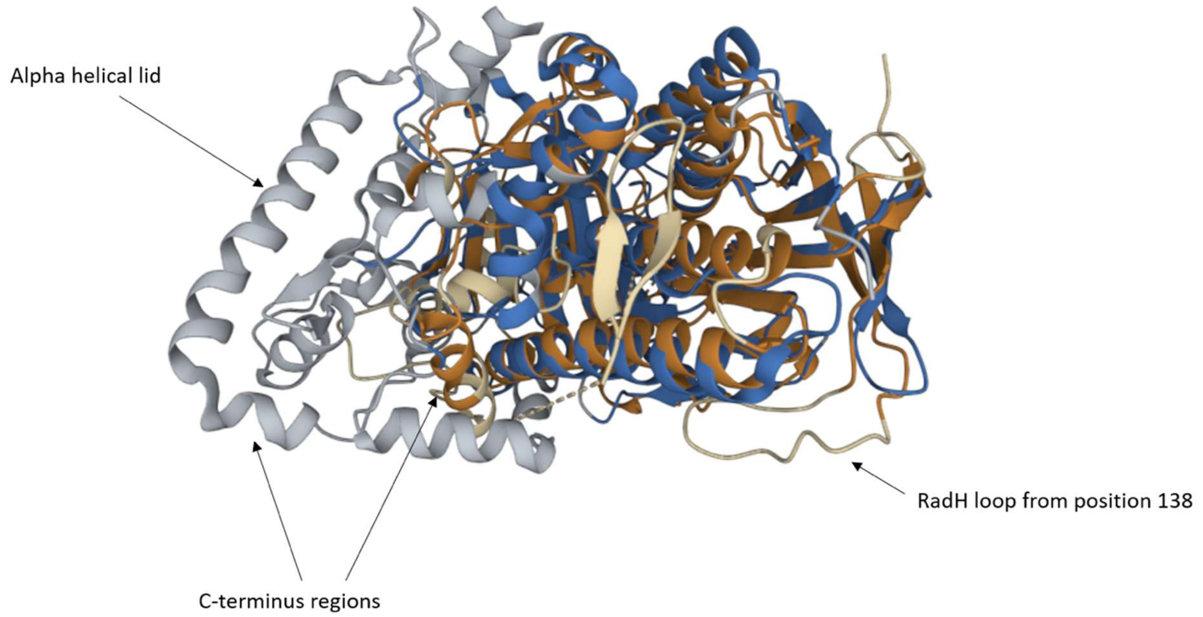

Figure S7. Structural alignment of RadH with RebH. RadH and RebH denoted in orange and blue, respectively. Structural features of interest for RadH and RebH are shown in cream and grey, respectively.

d) LC-MS analytical data for substrates panel using purified RadH

1-Cl

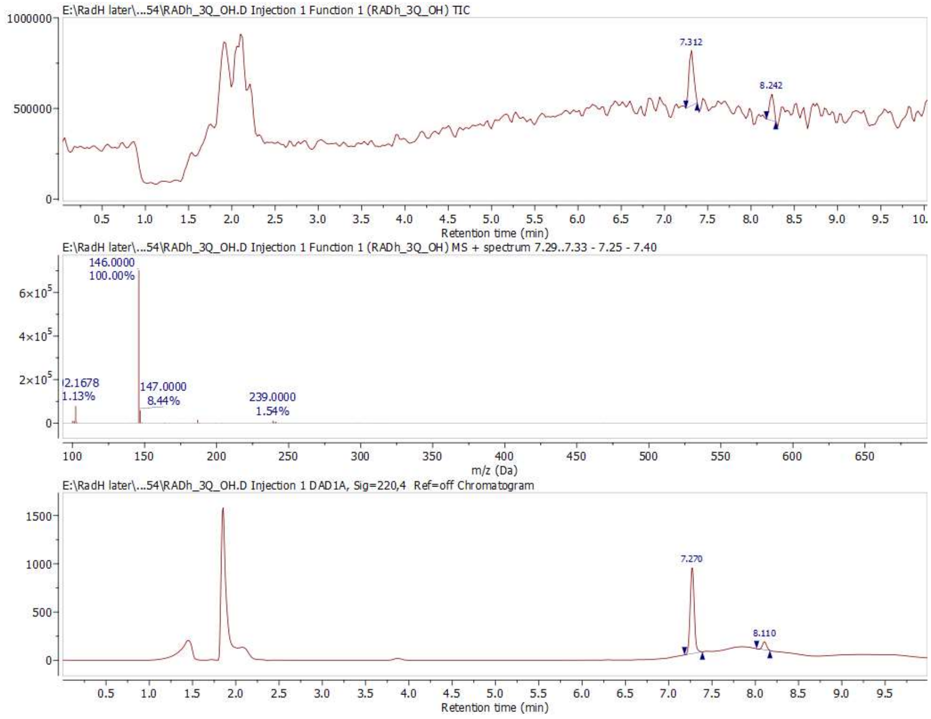

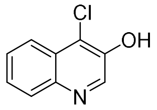

Chemical Formula: C_9H_6ClNO
Exact Mass: 179.0138

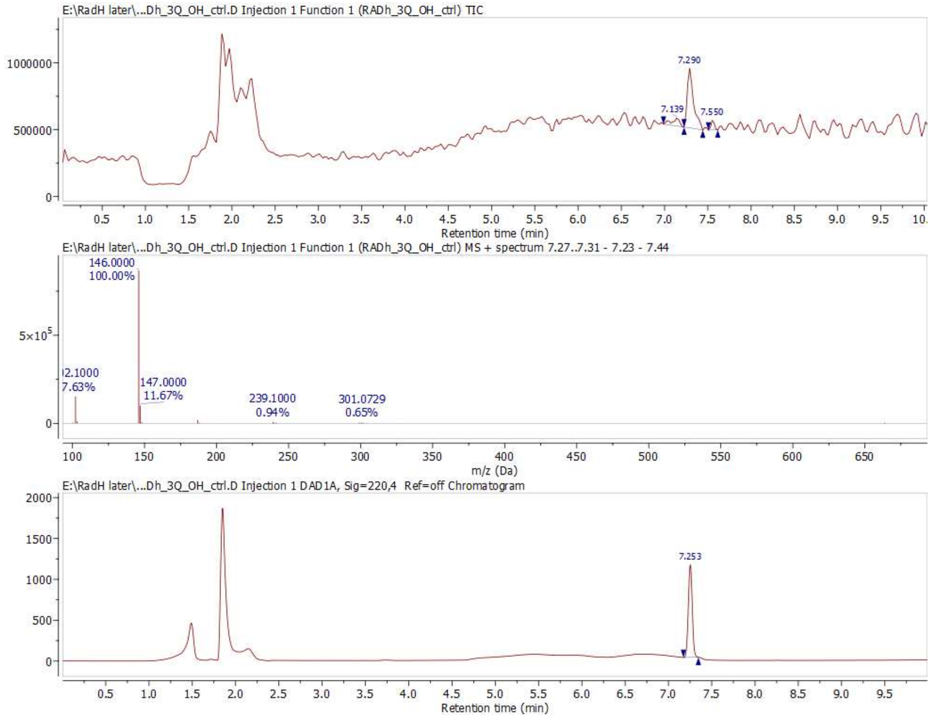

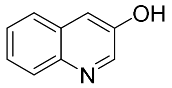

Chemical Formula: C_9H_7NO
Exact Mass: 145.0528

Figure S8. LCMS chromatogram for enzymatic reaction and its control for product 1-Cl.

2-Cl

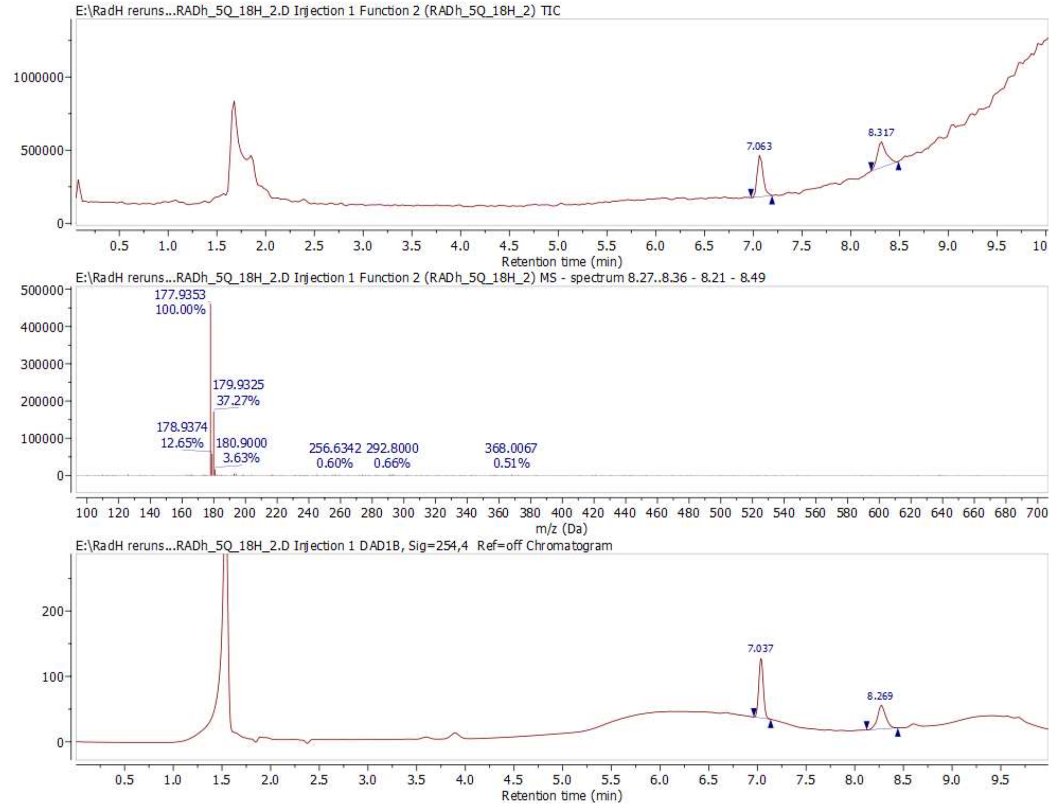

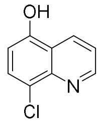

Chemical Formula: C_9H_6ClNO
Exact Mass: 179.0138

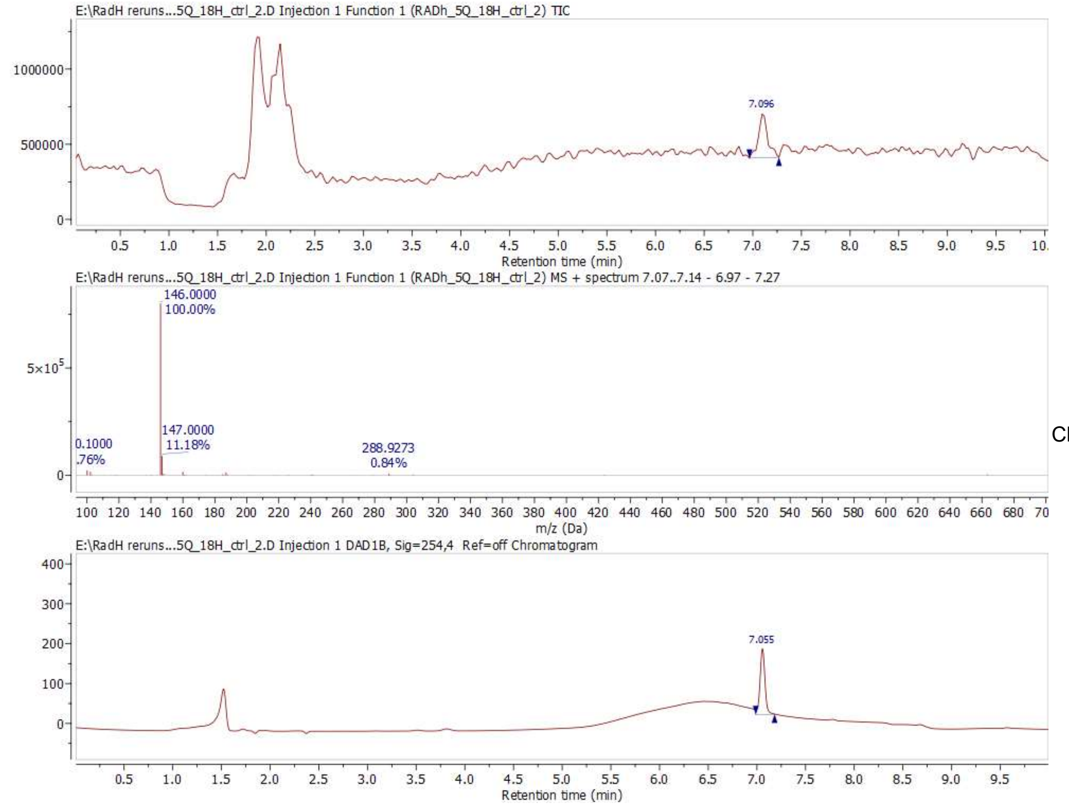

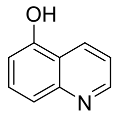

Chemical Formula: C_9H_7NO
Exact Mass: 145.0528

Figure S9. LCMS chromatogram for enzymatic reaction and its control for product 2-Cl.

3-Cl

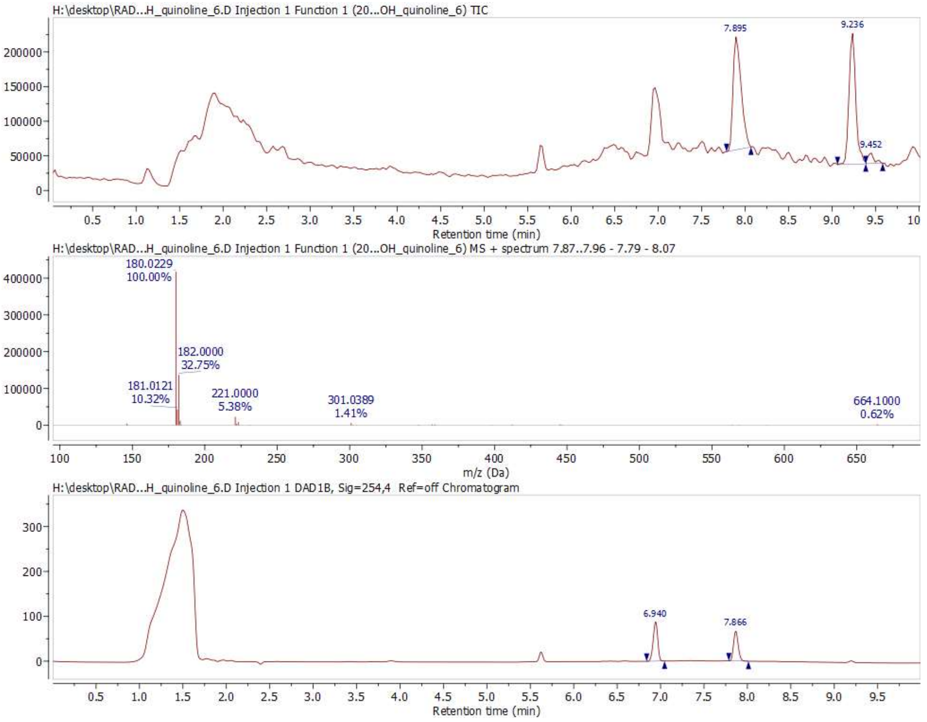

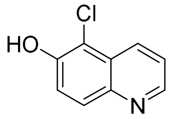

Chemical Formula: C_9H_6ClNO
Exact Mass: 179.0138

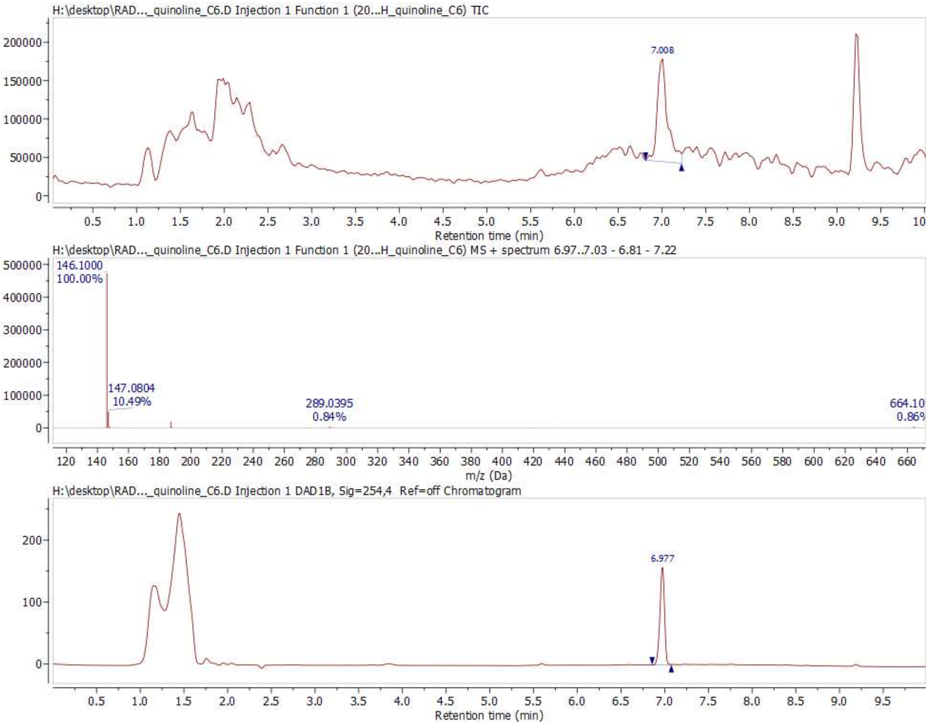

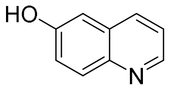

Chemical Formula: C_9H_7NO
Exact Mass: 145.0528

Figure S10. LCMS chromatogram for enzymatic reaction and its control for product 3-Cl.

4-Cl

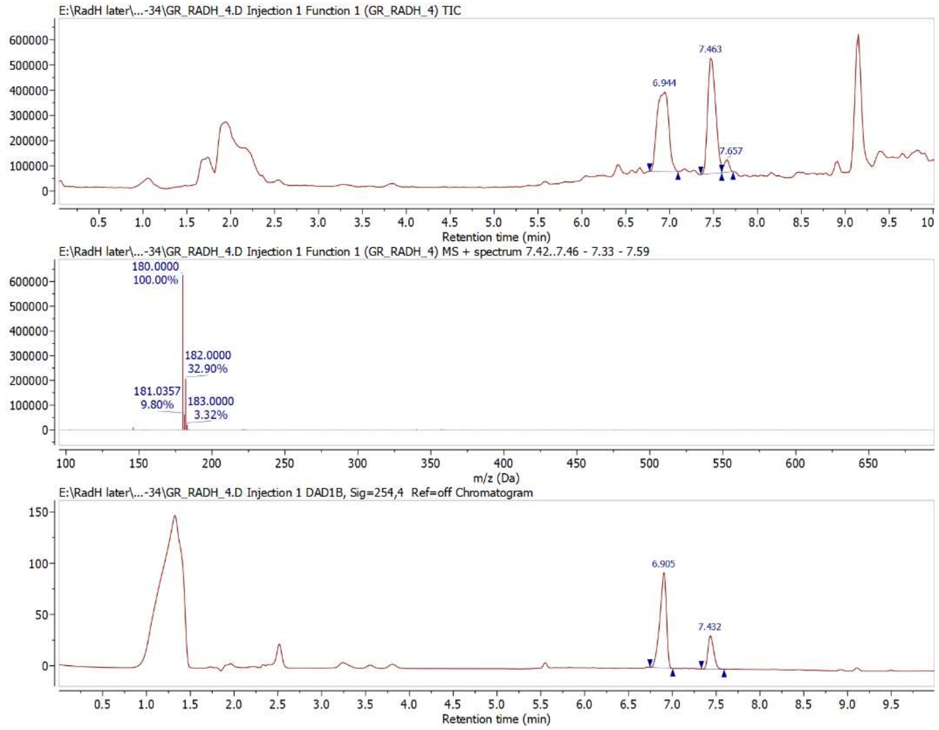

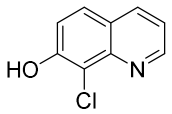

Chemical Formula: C_9H_6ClNO
Exact Mass: 179.0138

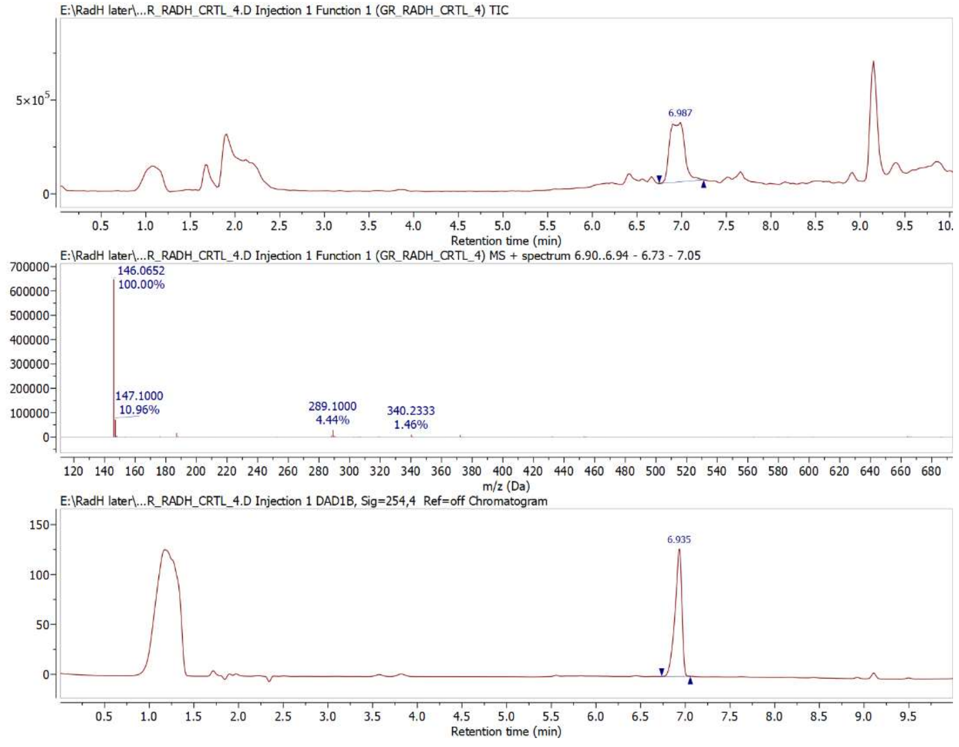

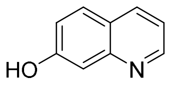

Chemical Formula: C_9H_7NO
Exact Mass: 145.0528

Figure S11. LCMS chromatogram for enzymatic reaction and its control for product 4-Cl.

19-Cl

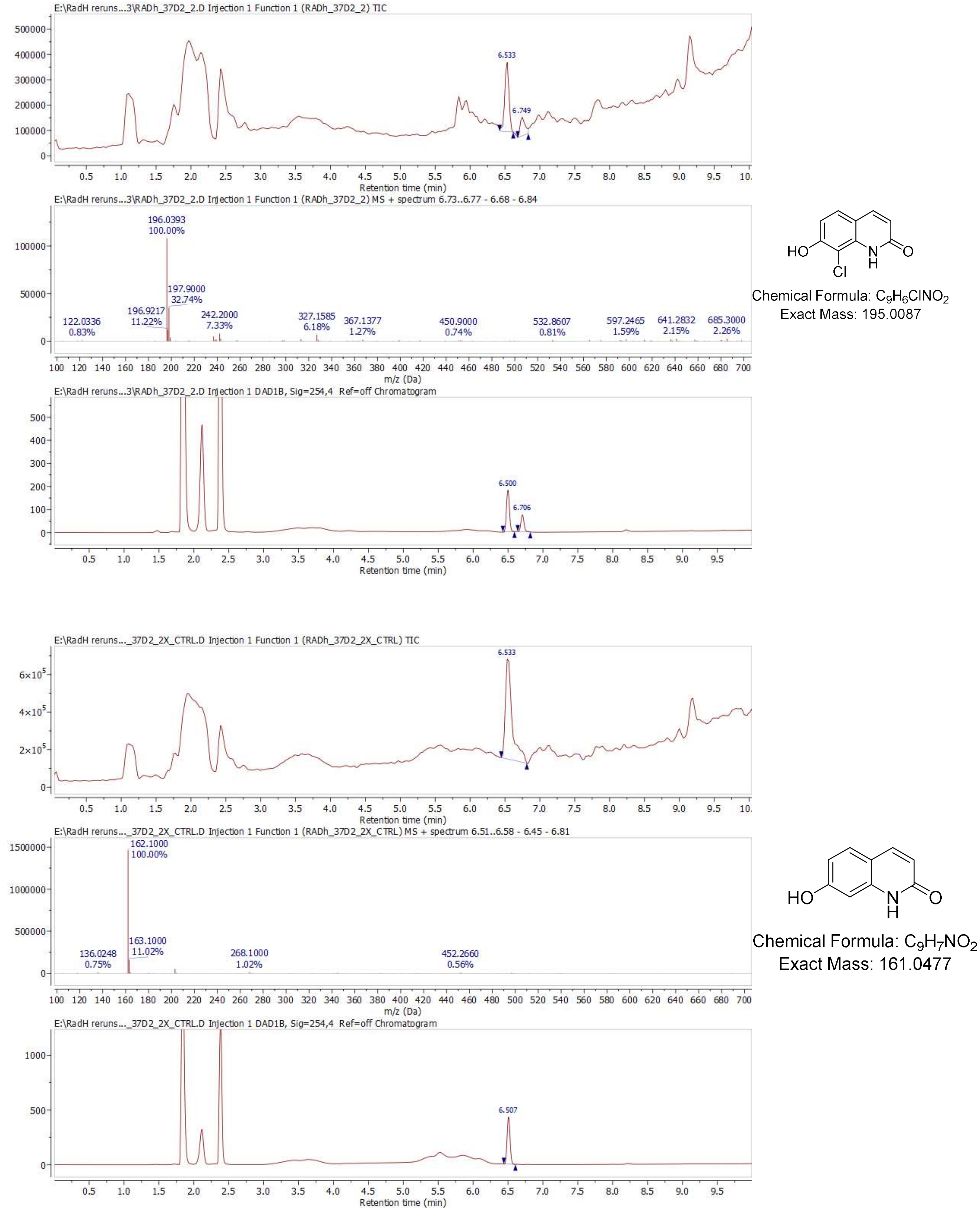

Figure S12. LCMS chromatogram for enzymatic reaction and its control for product 19-Cl.

22-Cl

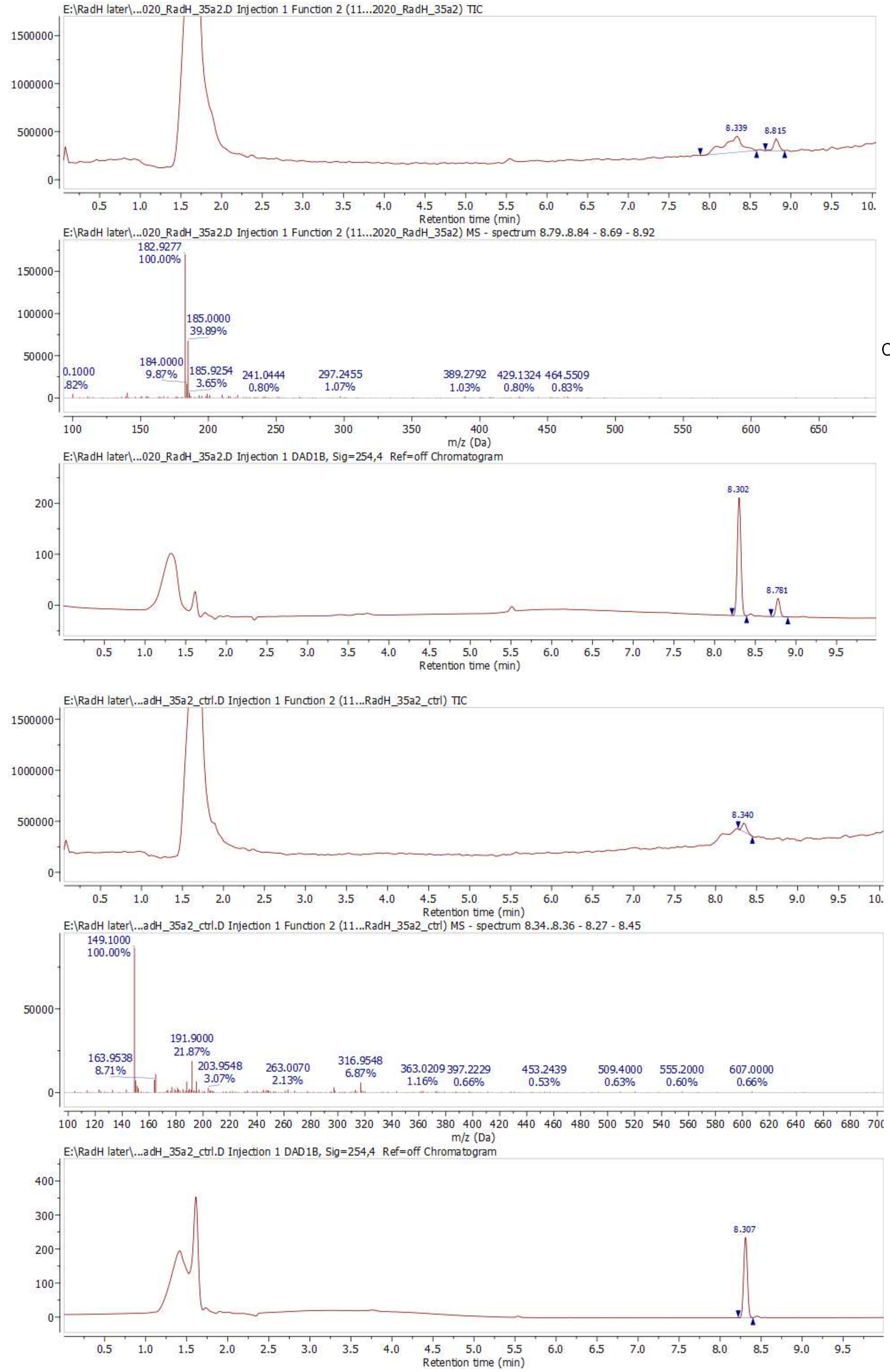

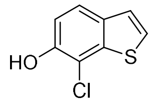

Chemical Formula: C_8H_5ClOS
Exact Mass: 183.9750

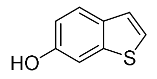

Chemical Formula: C_8H_6OS
Exact Mass: 150.0139

Figure S13. LCMS chromatogram for enzymatic reaction and its control for product 22-Cl.

c) NMR Spectra of selected halogenated products

1-Cl

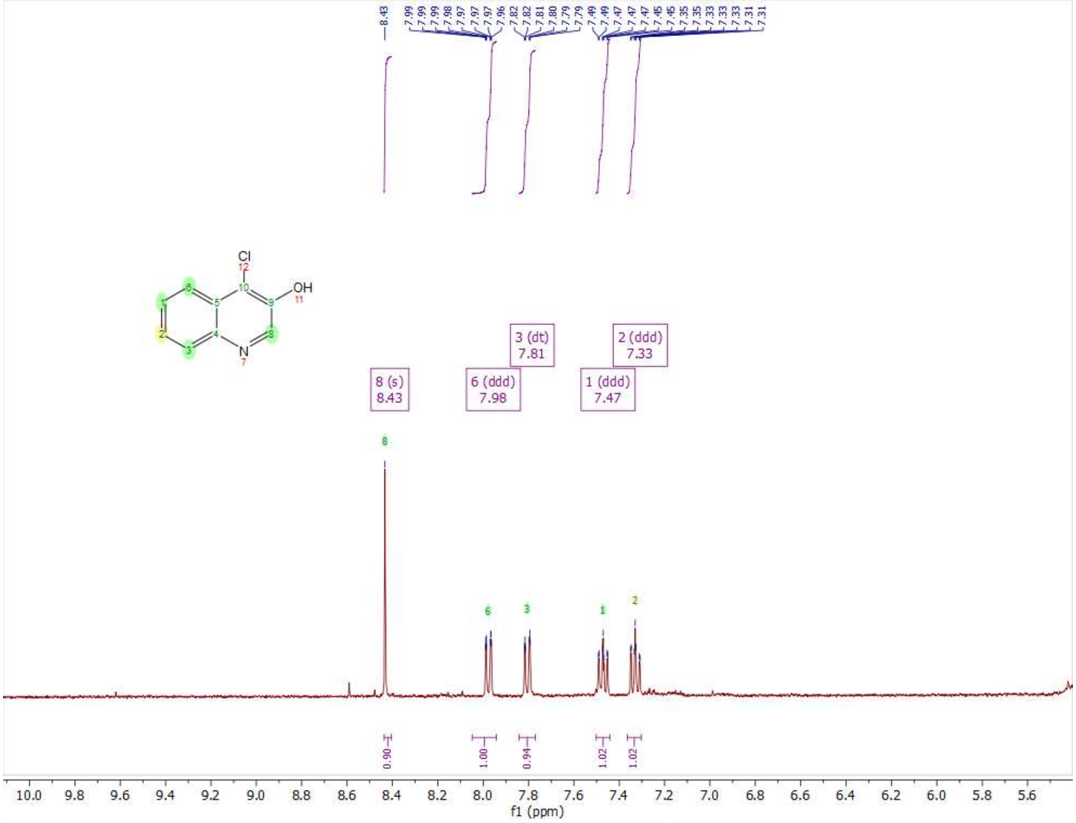

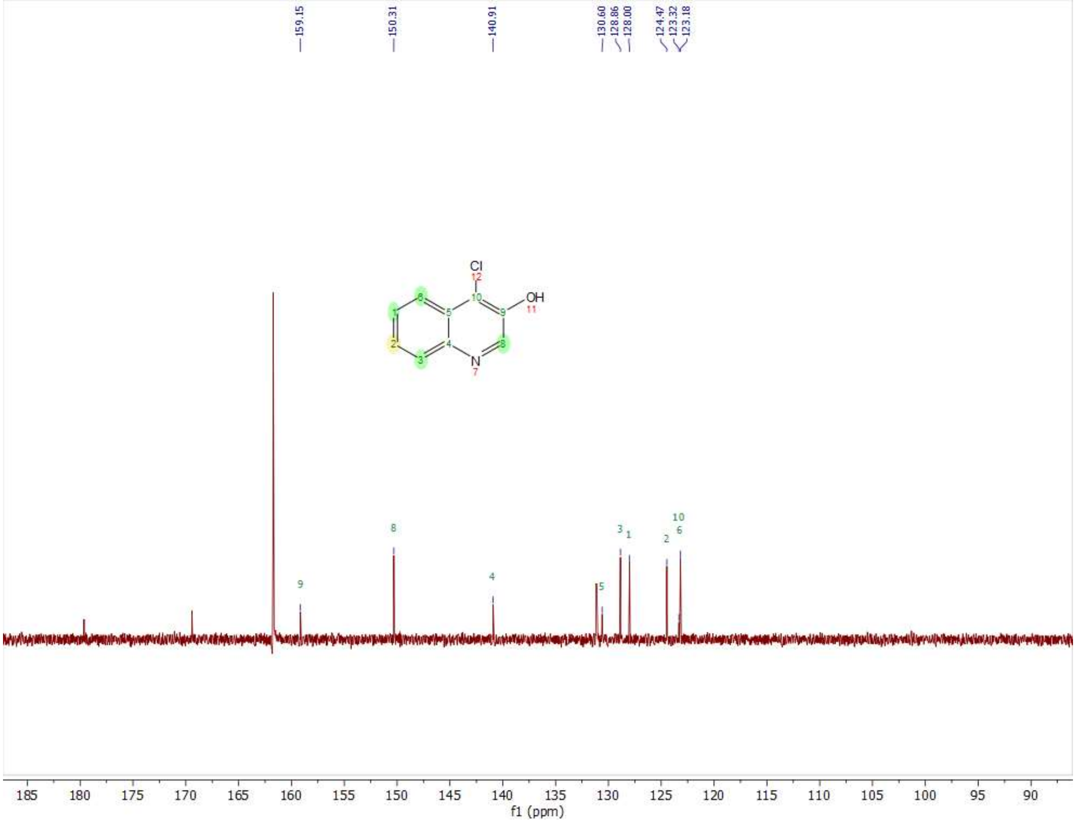

Figure S14. ^{1}H and ^{13}C NMR spectrum for 1-Cl.

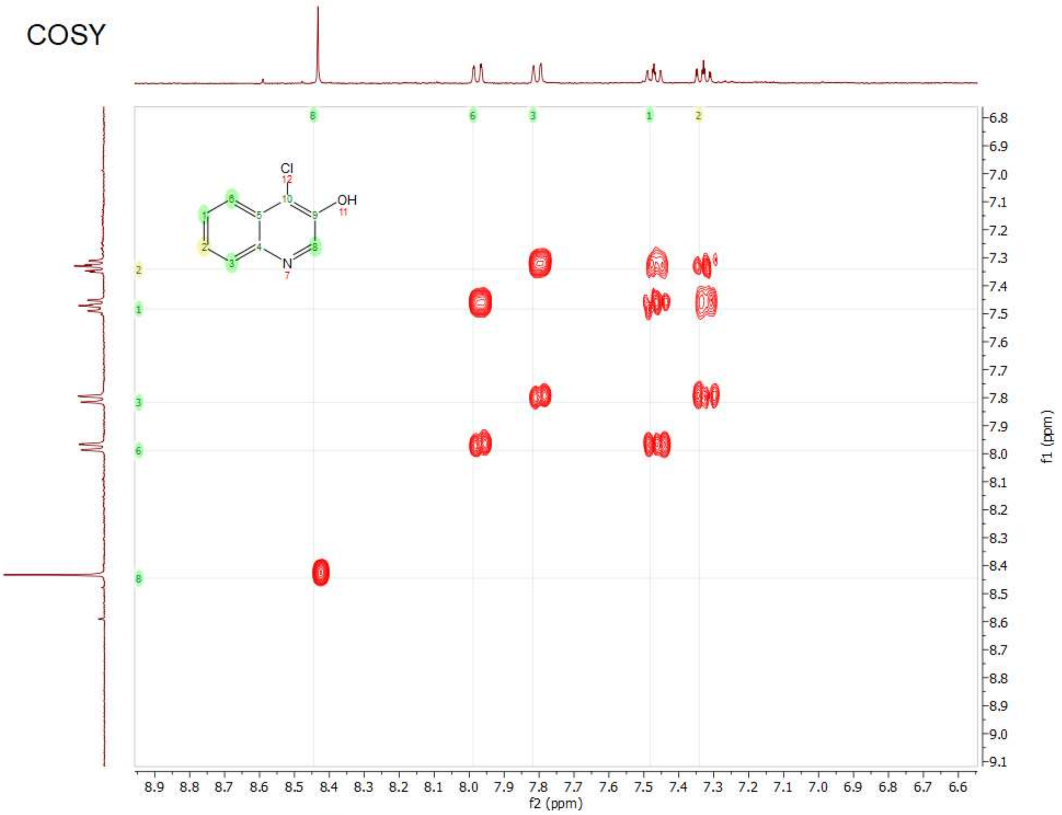

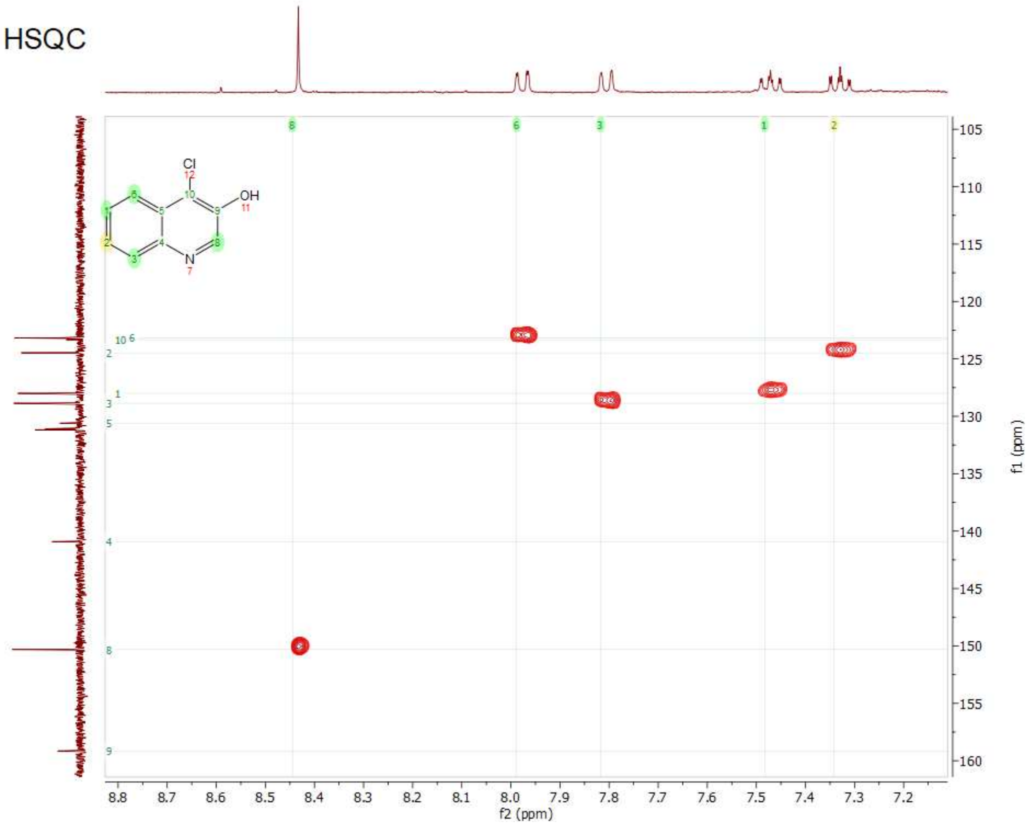

Figure S15. ^{1}H-^{1}H COSY and ^{1}H-^{13}C HSQC spectrum for 1-Cl.

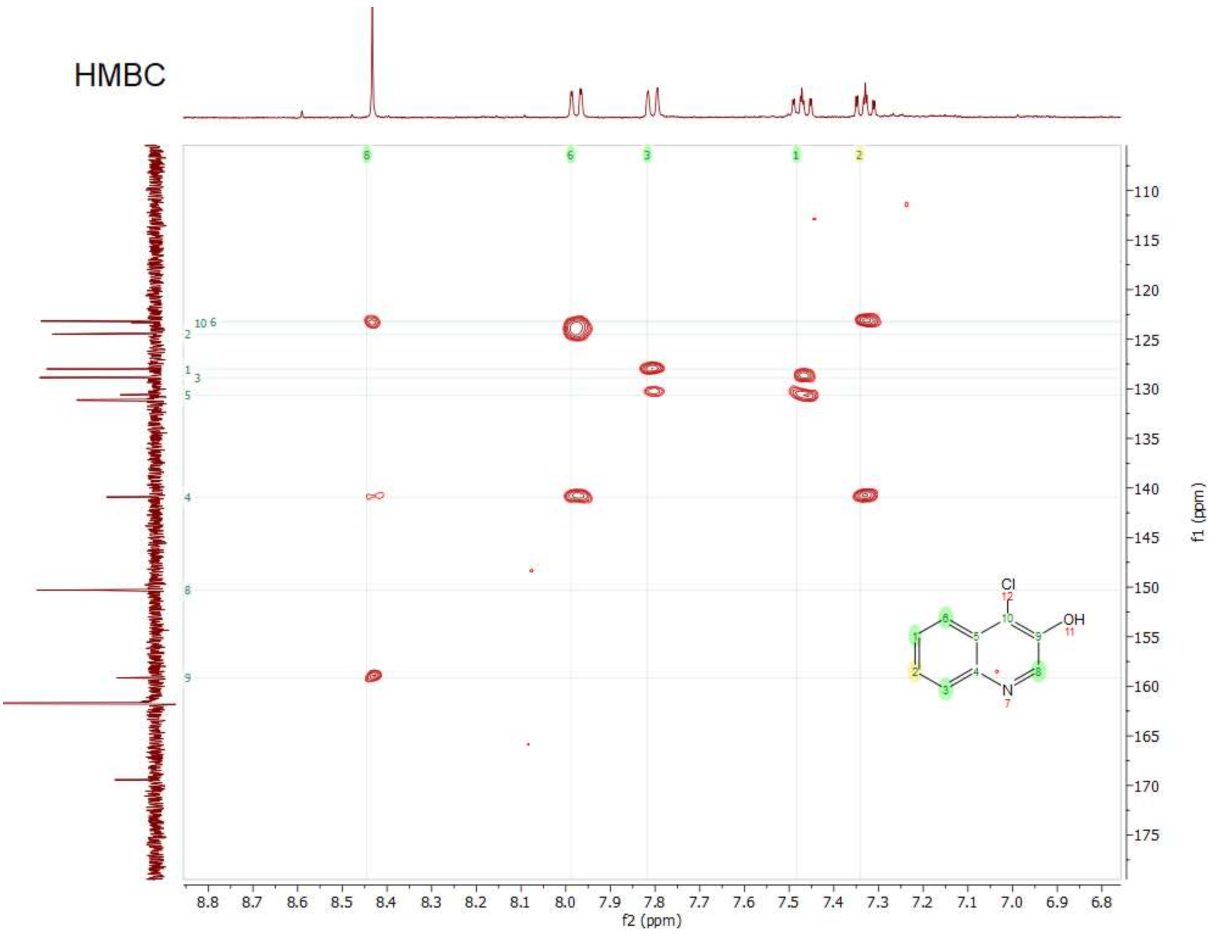

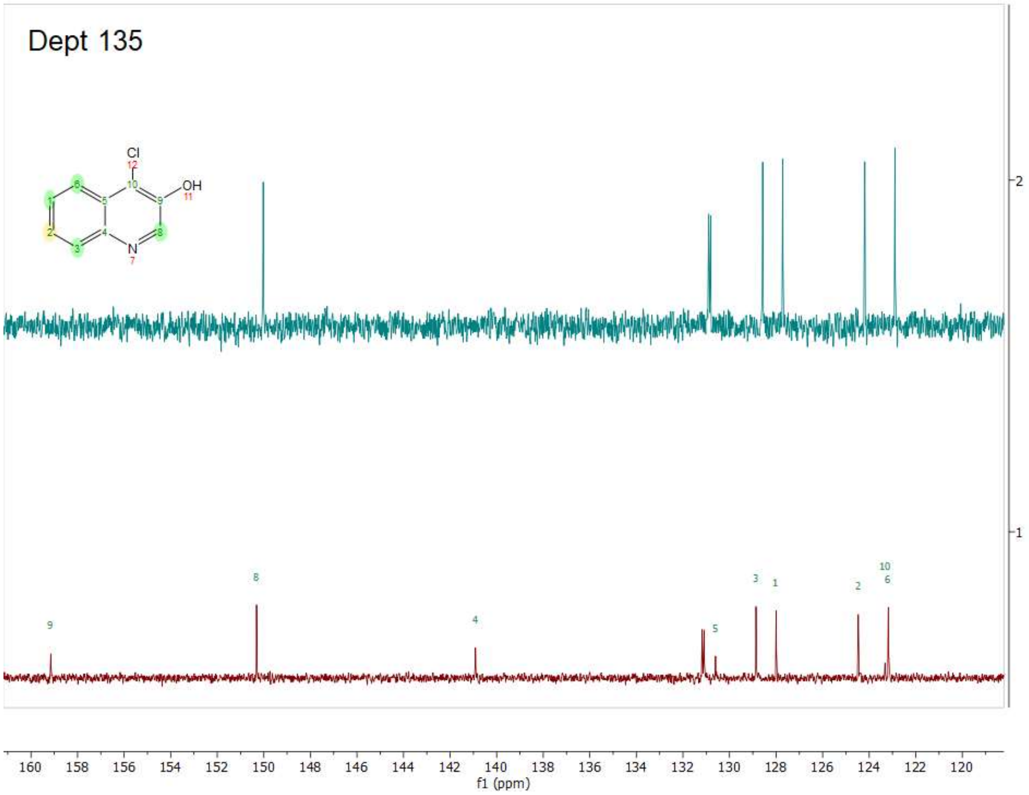

Figure S16. ^{1}H-^{13}C HMBC and ^{13}C DEPT135 spectrum for 1-Cl.

2-Cl

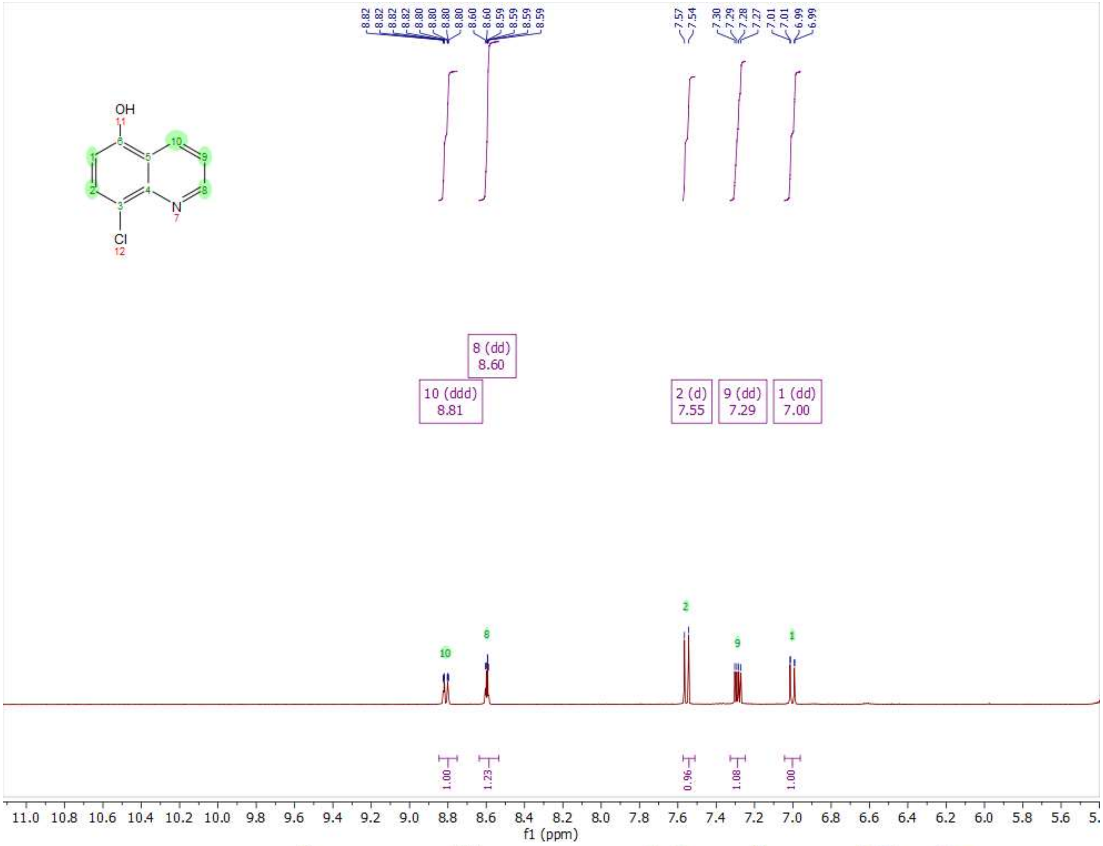

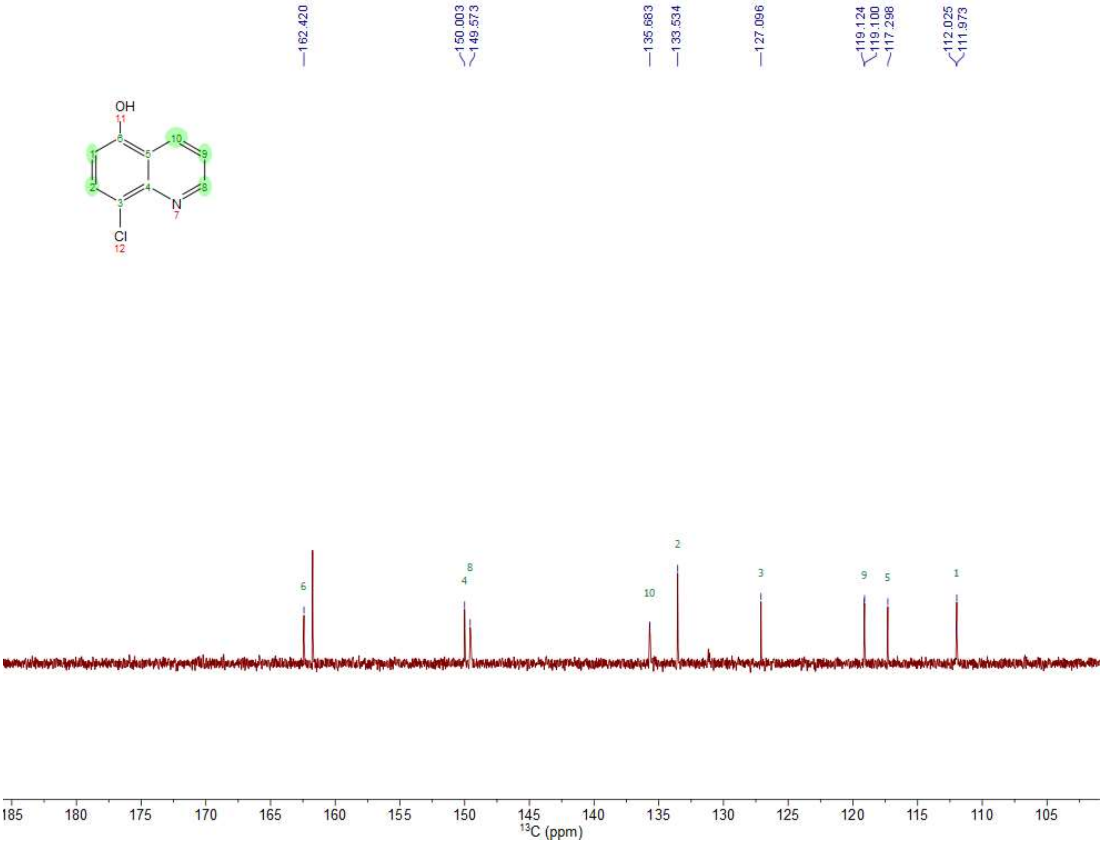

Figure S17. ^{1}H and ^{13}C NMR spectrum for 2-Cl.

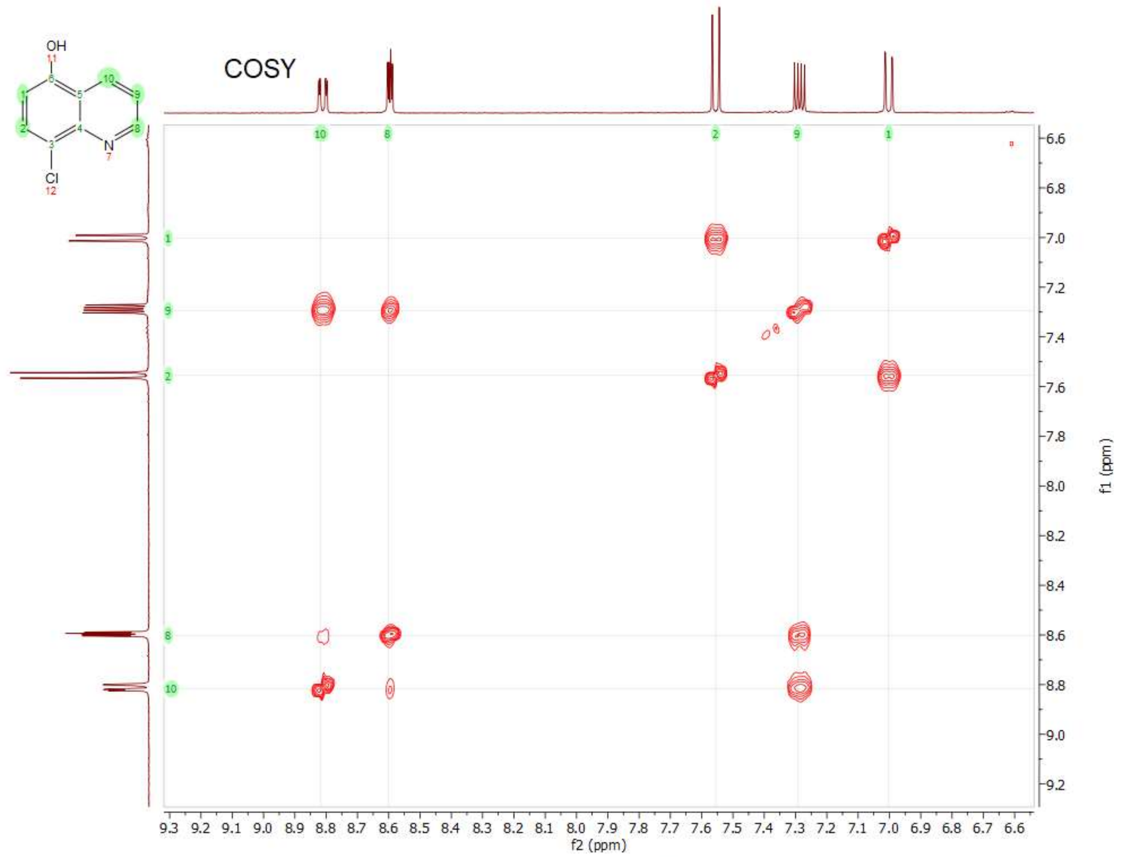

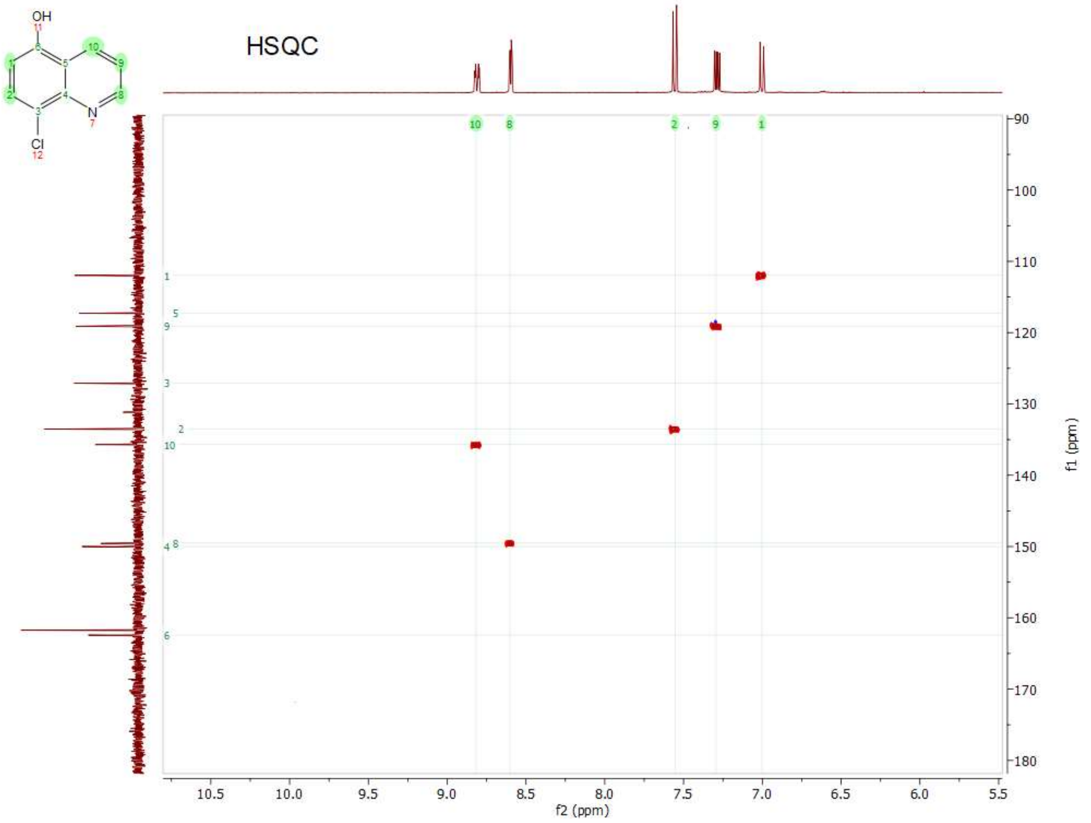

Figure S18. ^{1}H-^{1}H COSY and ^{1}H-^{13}C HSQC spectrum for 2-Cl.

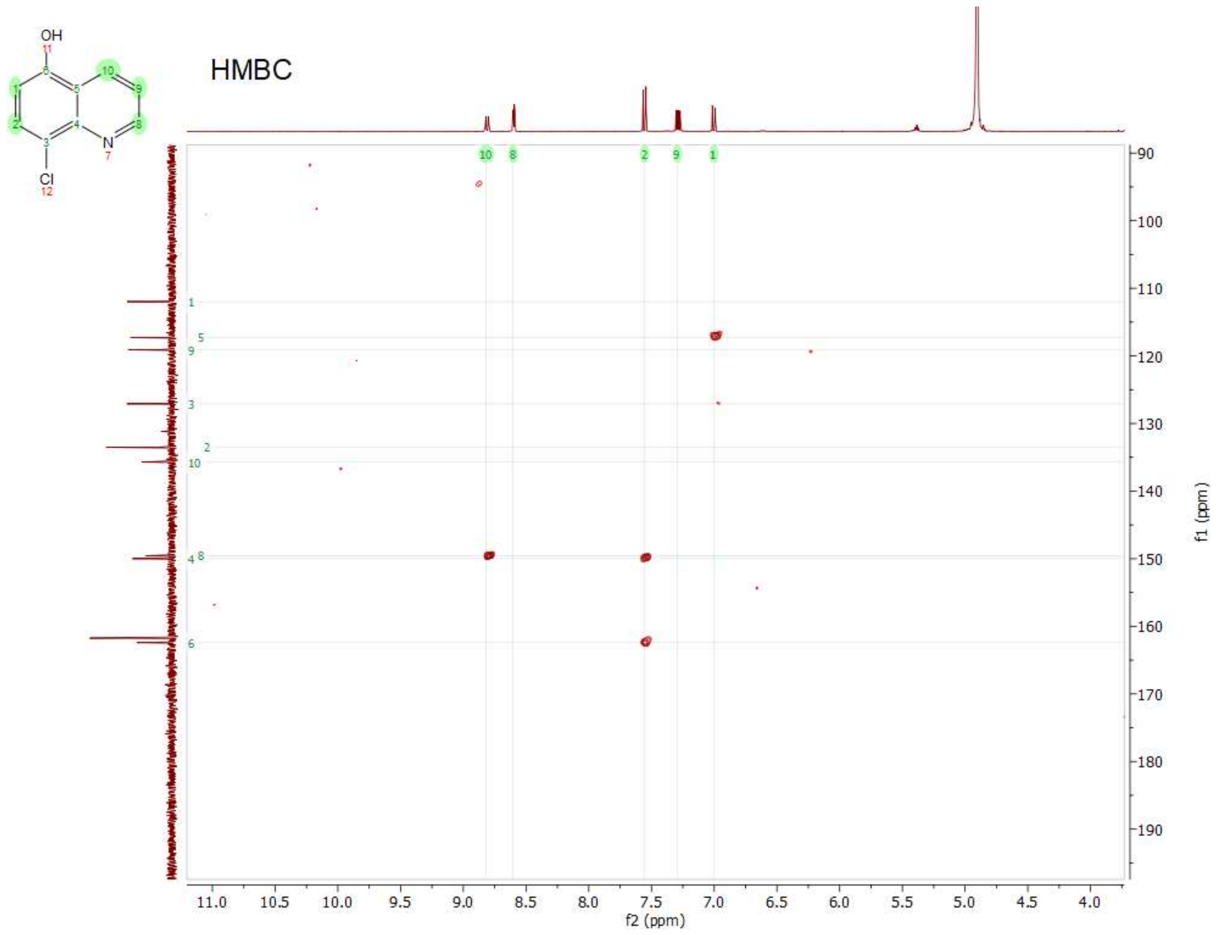

Figure S19. ^{1}H -^{13}C HMBC spectrum for 2-Cl.

3-Cl

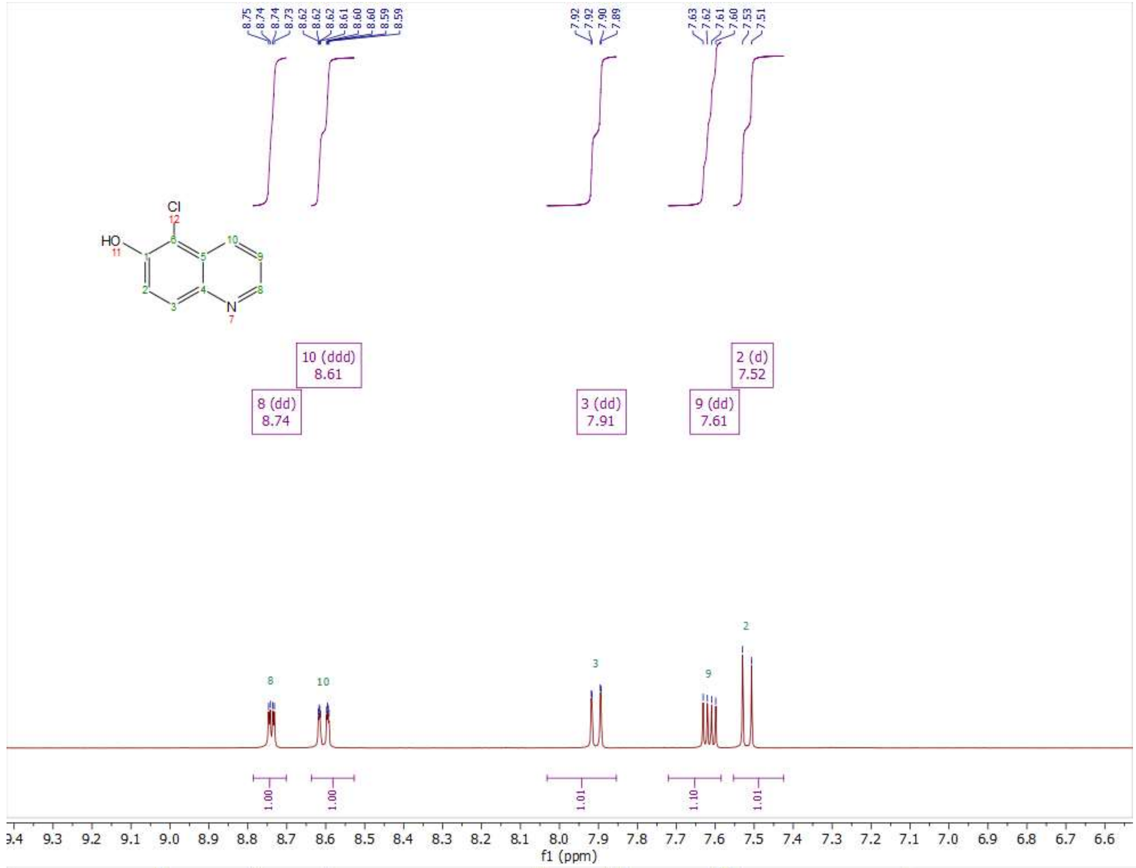

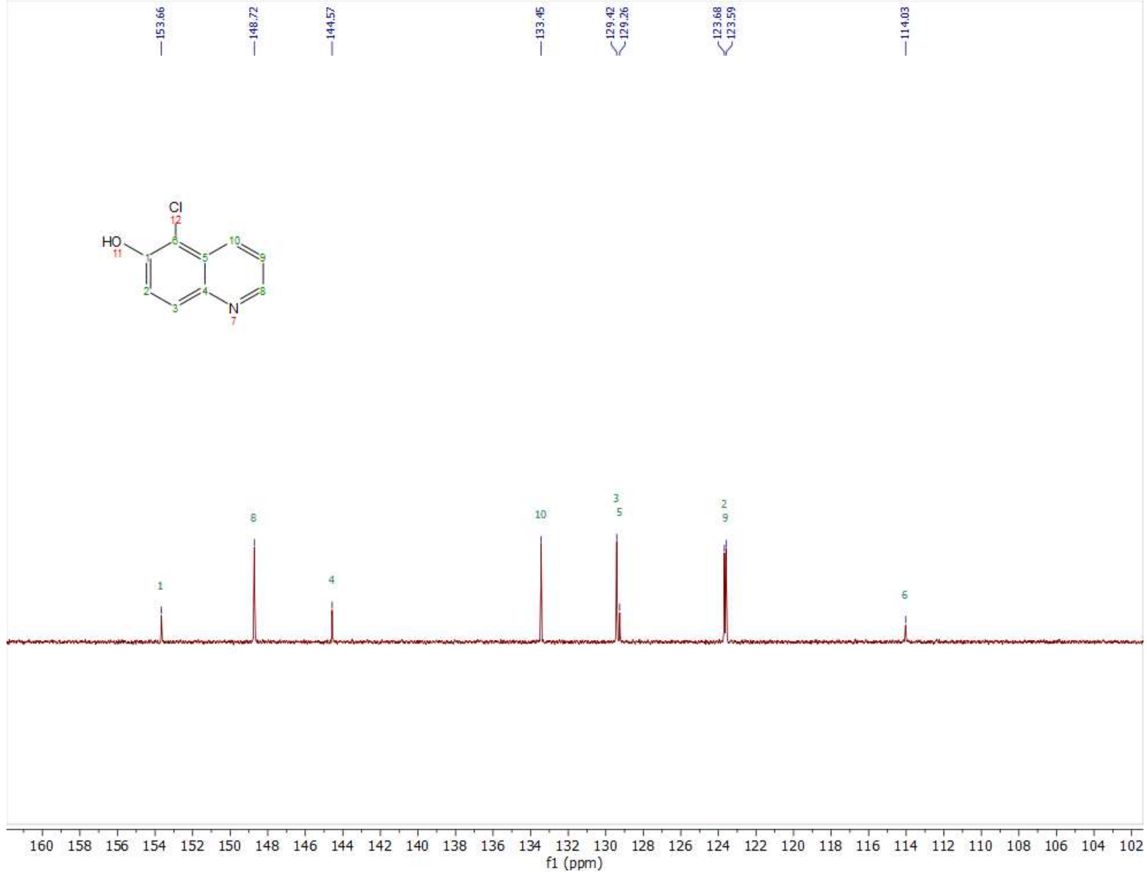

Figure S20. ¹H and ¹³C NMR spectrum for 3-Cl.

COSY

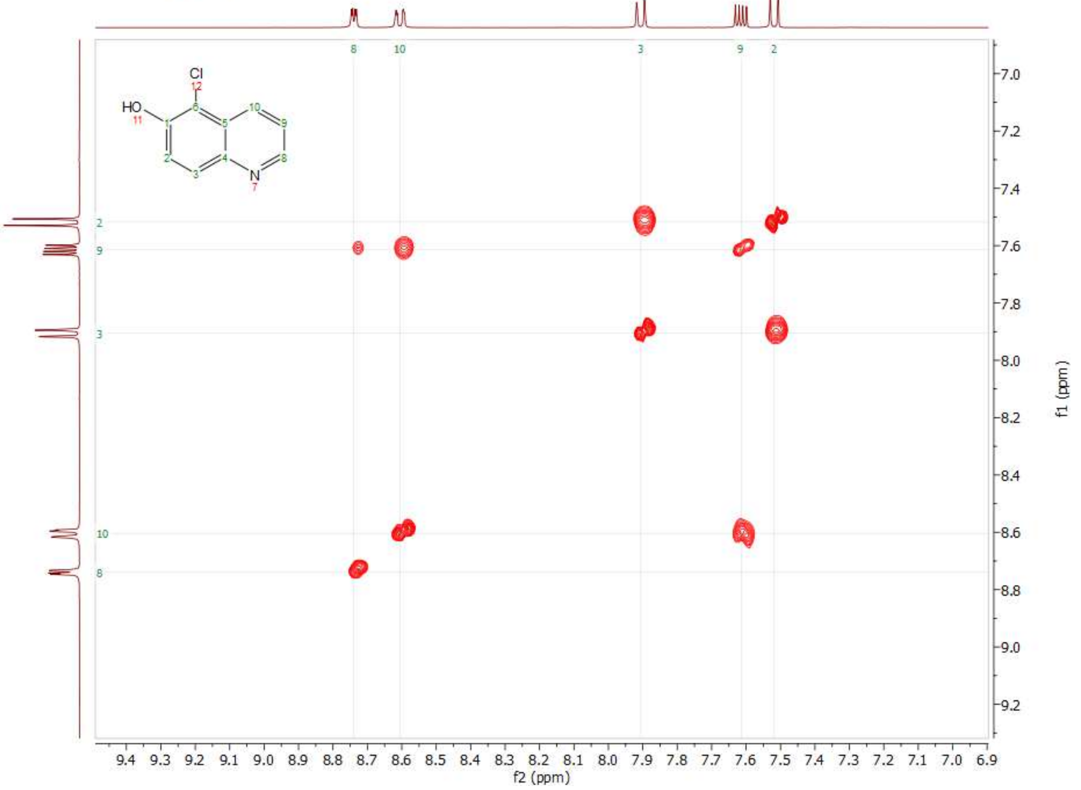

HMBC

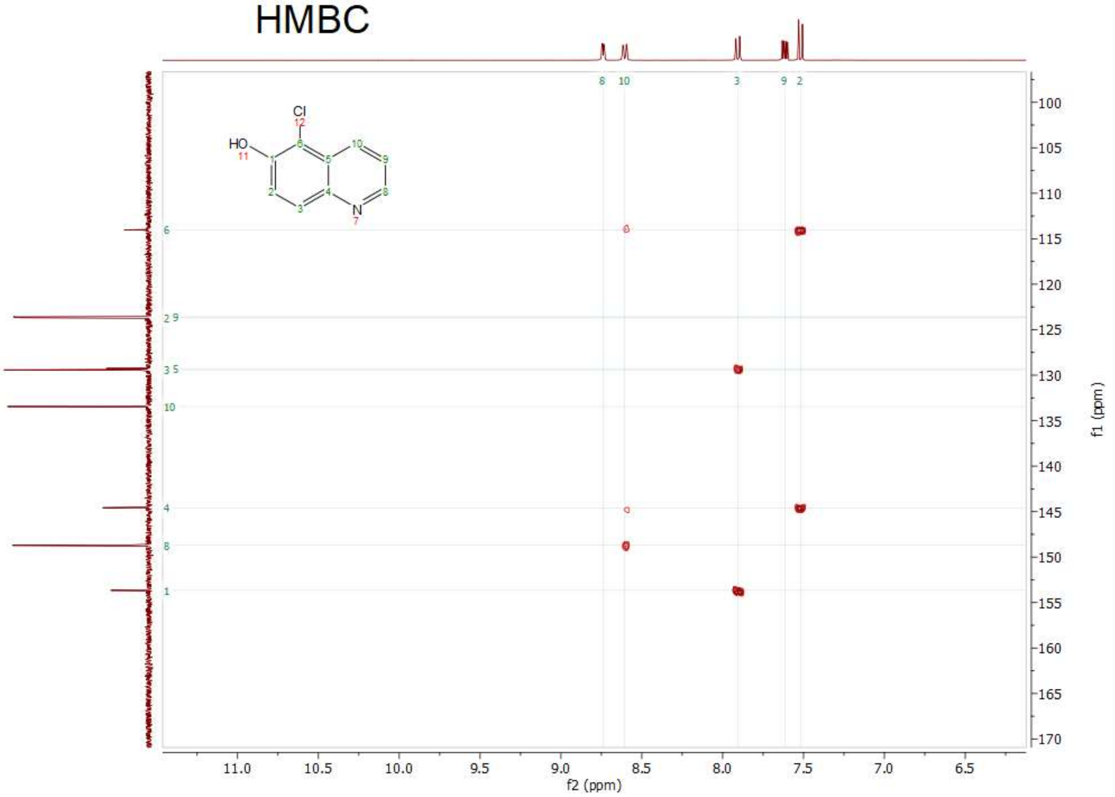

Figure S21. ^{1}H-^{1}H COSY and ^{1}H-^{13}C HMBC spectrum for 3-Cl.

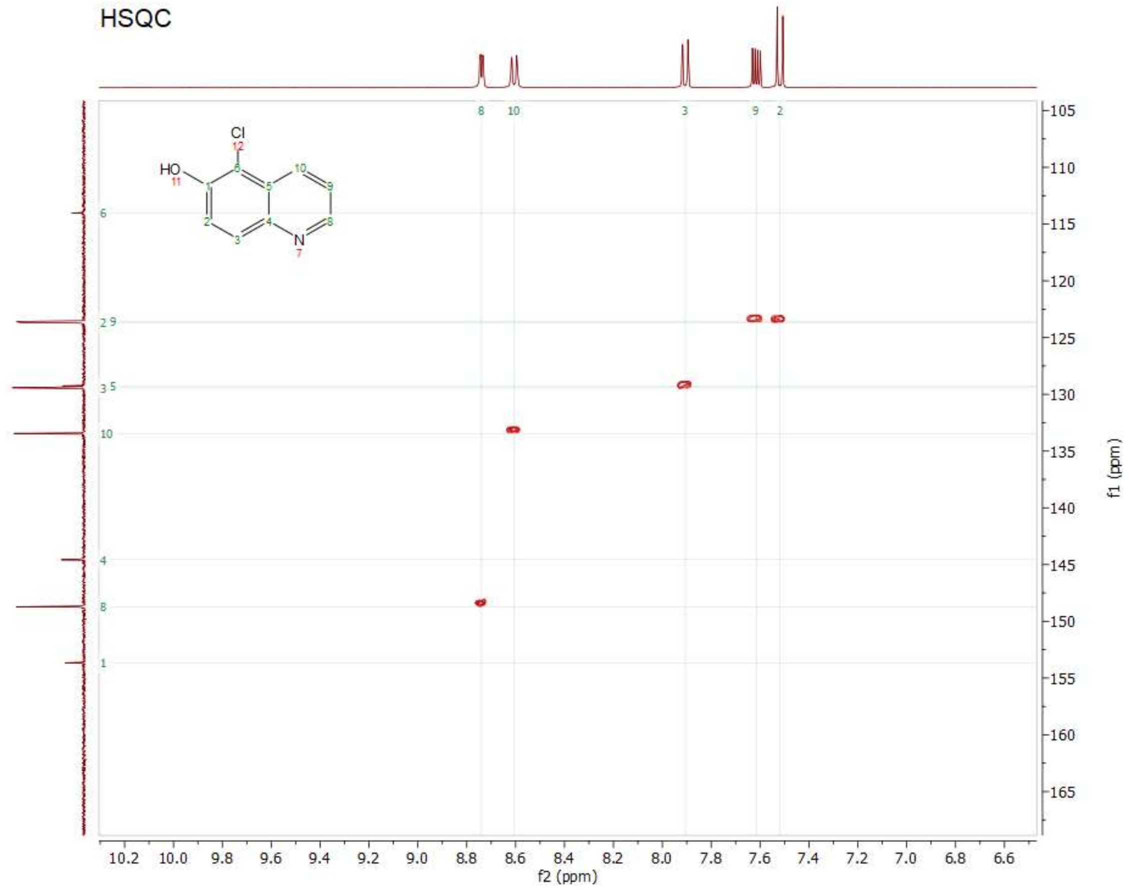

Figure S22. ^{1}H-^{13}C HSQC spectrum for 3-Cl.

4-Cl

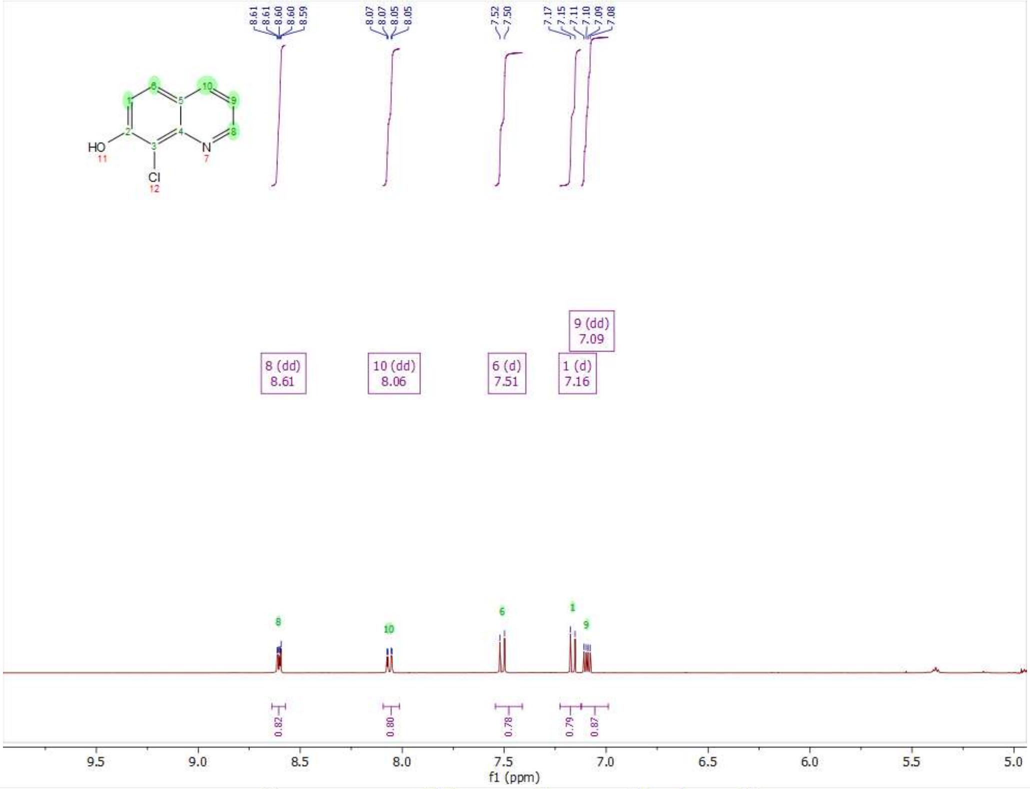

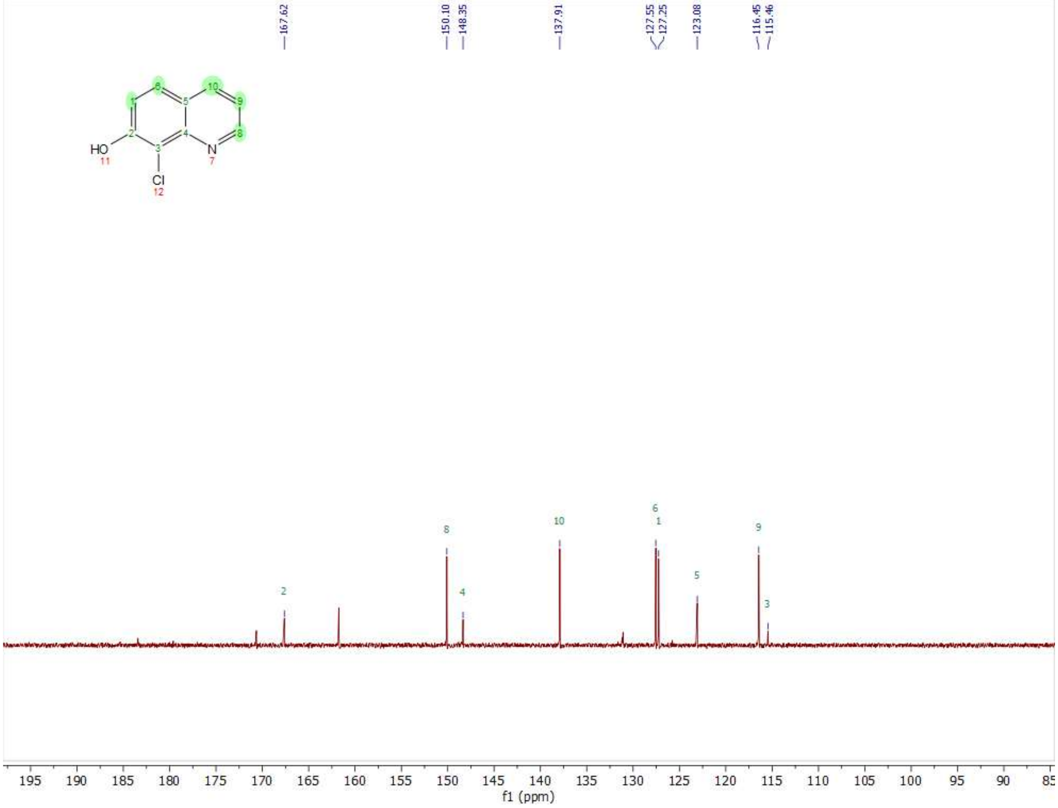

Figure S23. ^{1}H and ^{13}C NMR spectrum for 4-Cl.

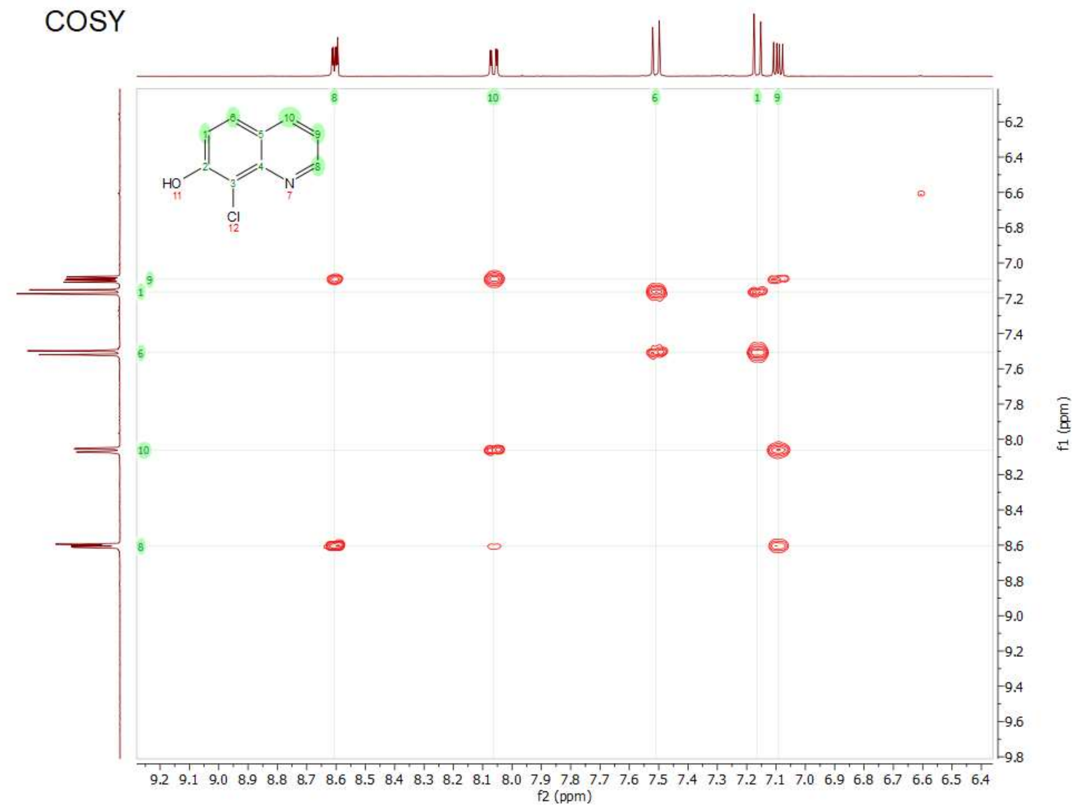

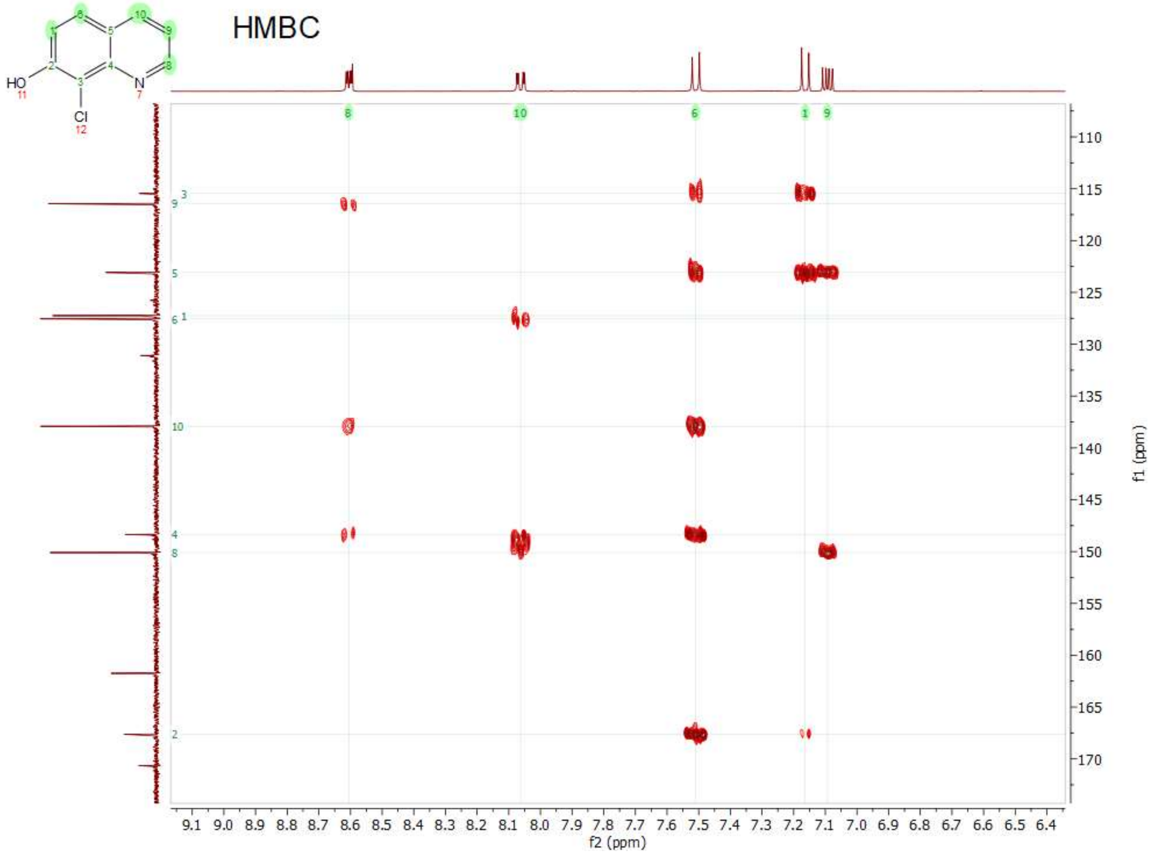

Figure S24. ^{1}H-^{1}H COSY and ^{1}H-^{13}C HMBC spectrum for 4-Cl.

HSQC

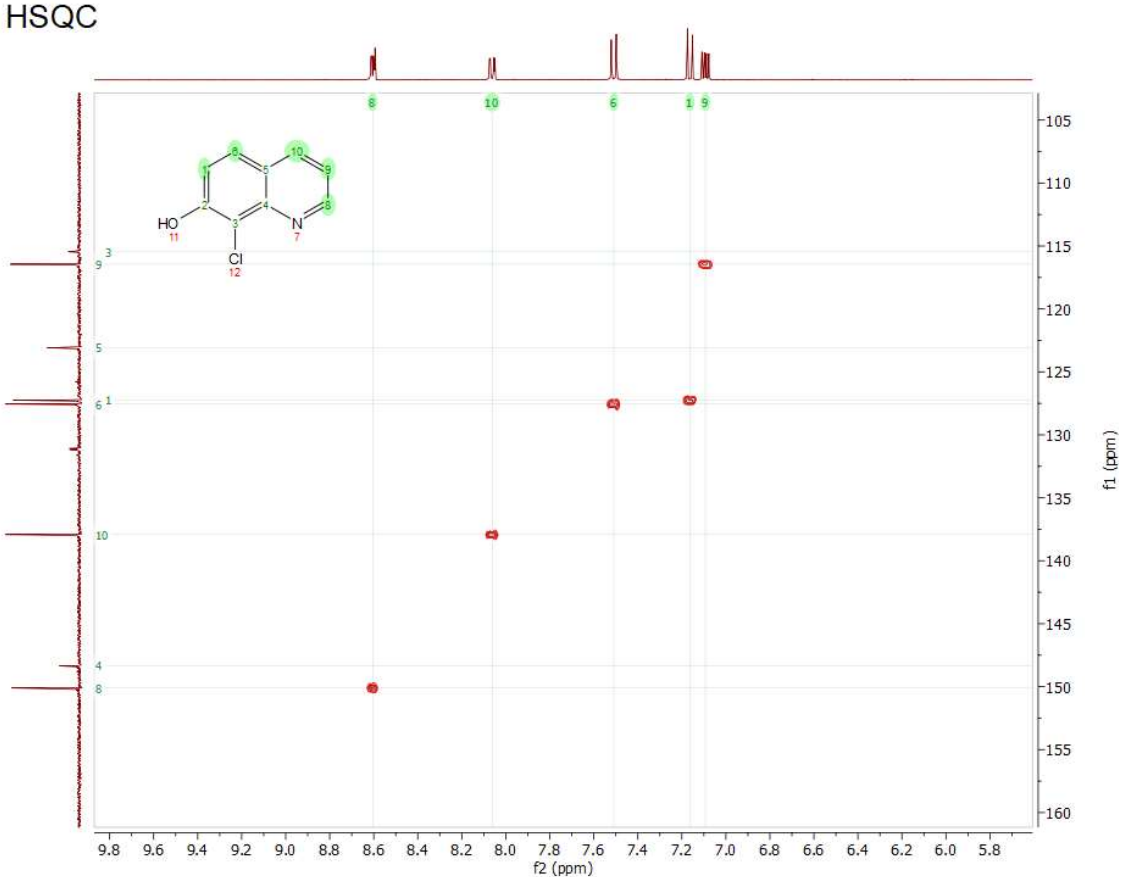

Figure S25. ^1H-^{13}C HSQC spectrum for 4-Cl.

19-Cl

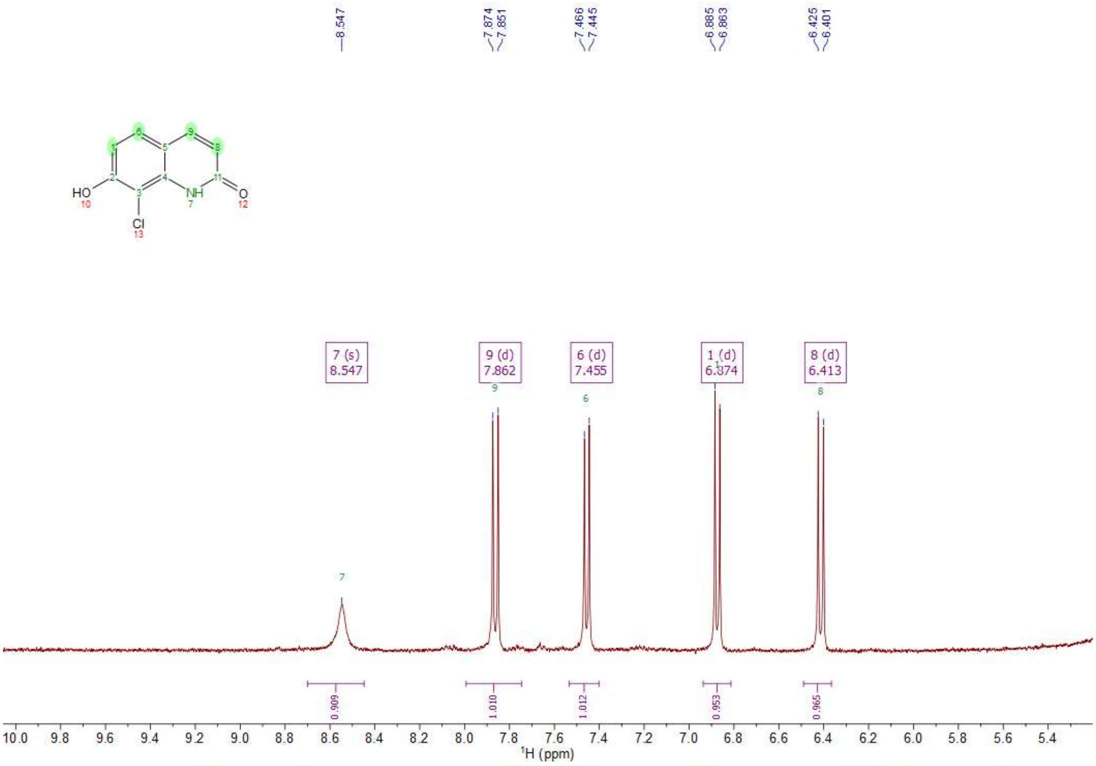

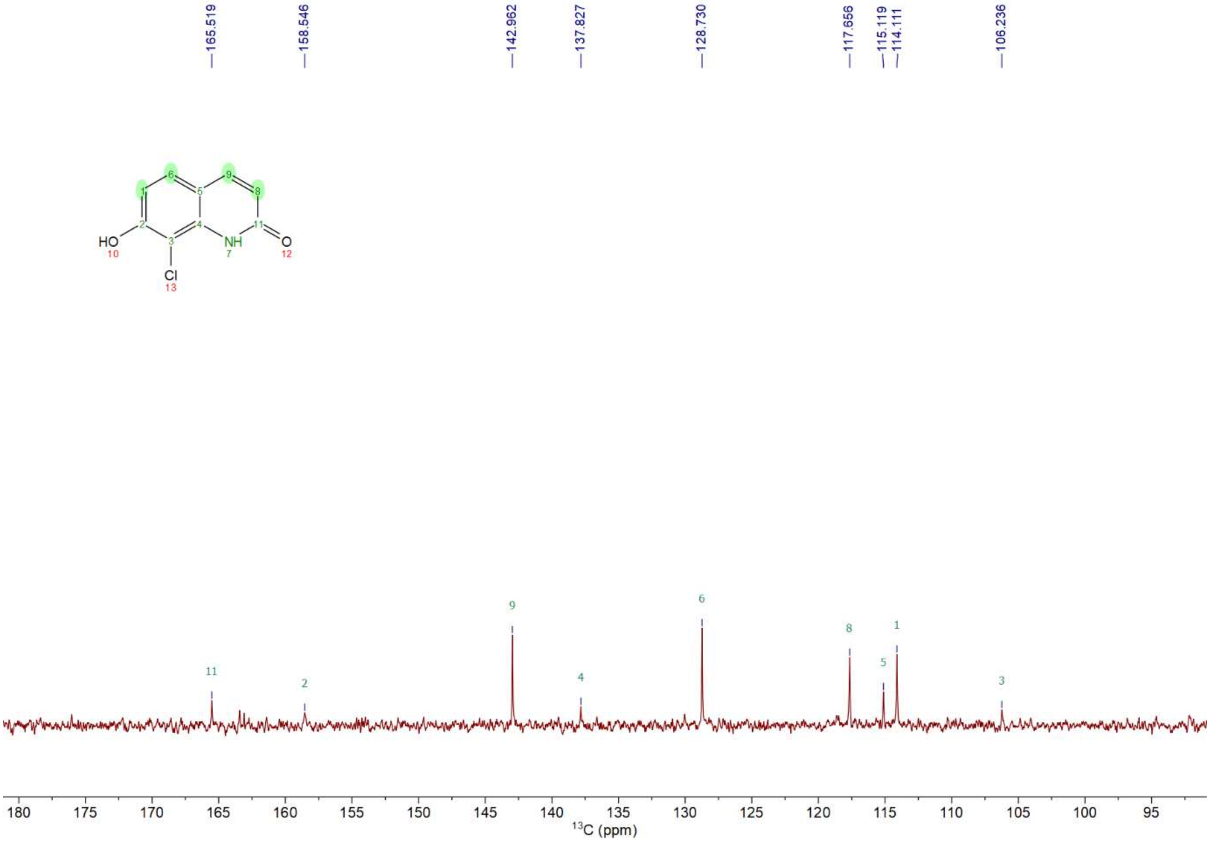

Figure S26. ^{1}H and ^{13}C NMR spectrum for 19-Cl.

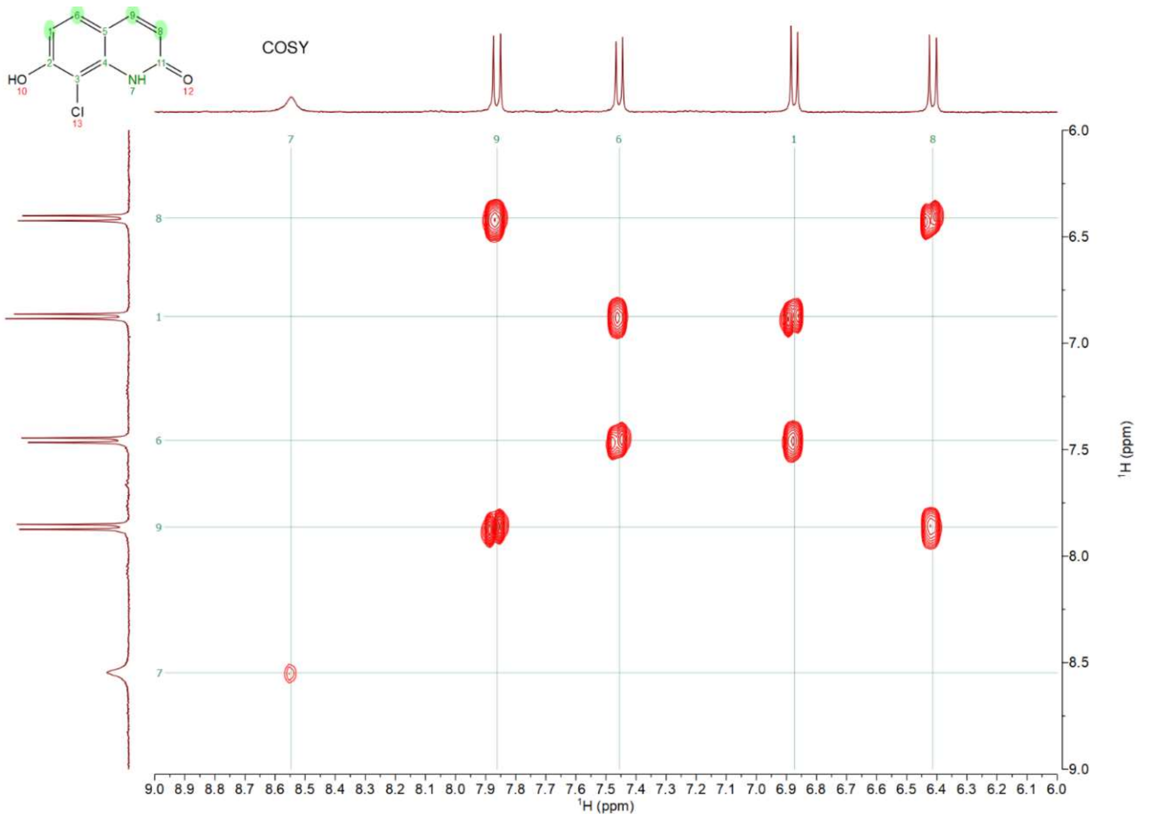

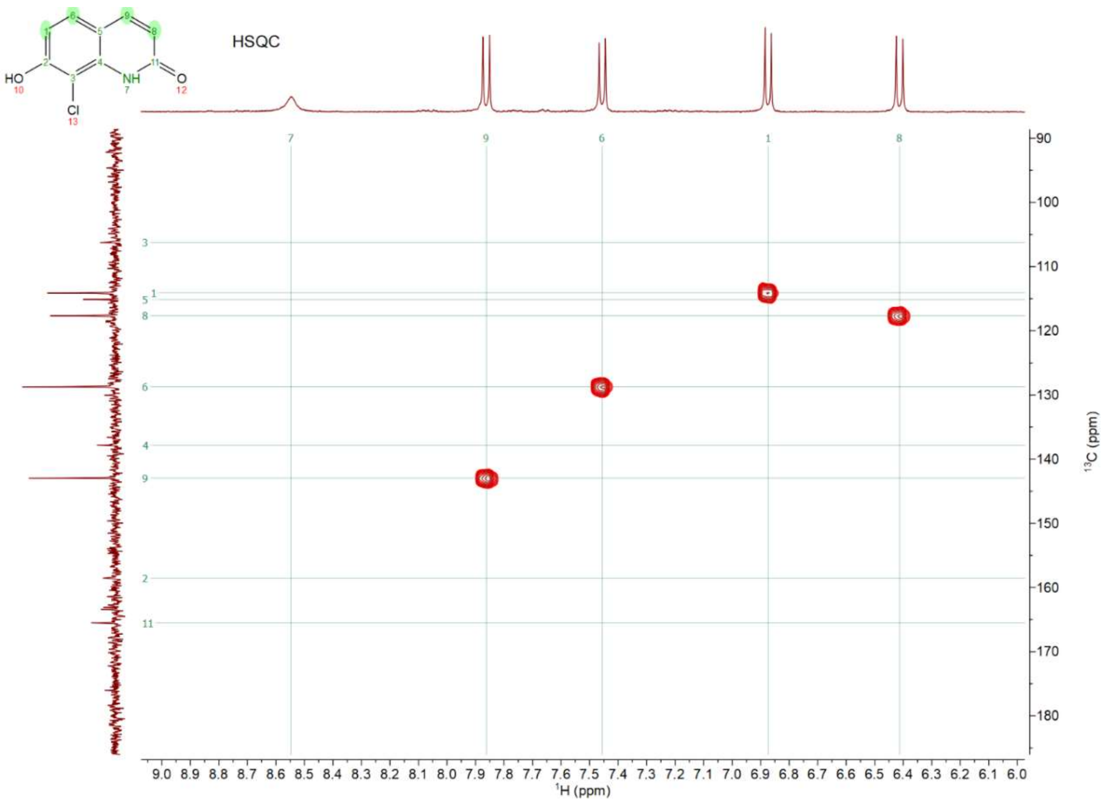

Figure S27. ^{1}H-^{1}H COSY and ^{1}H-^{13}C HSQC spectrum for 19-Cl.

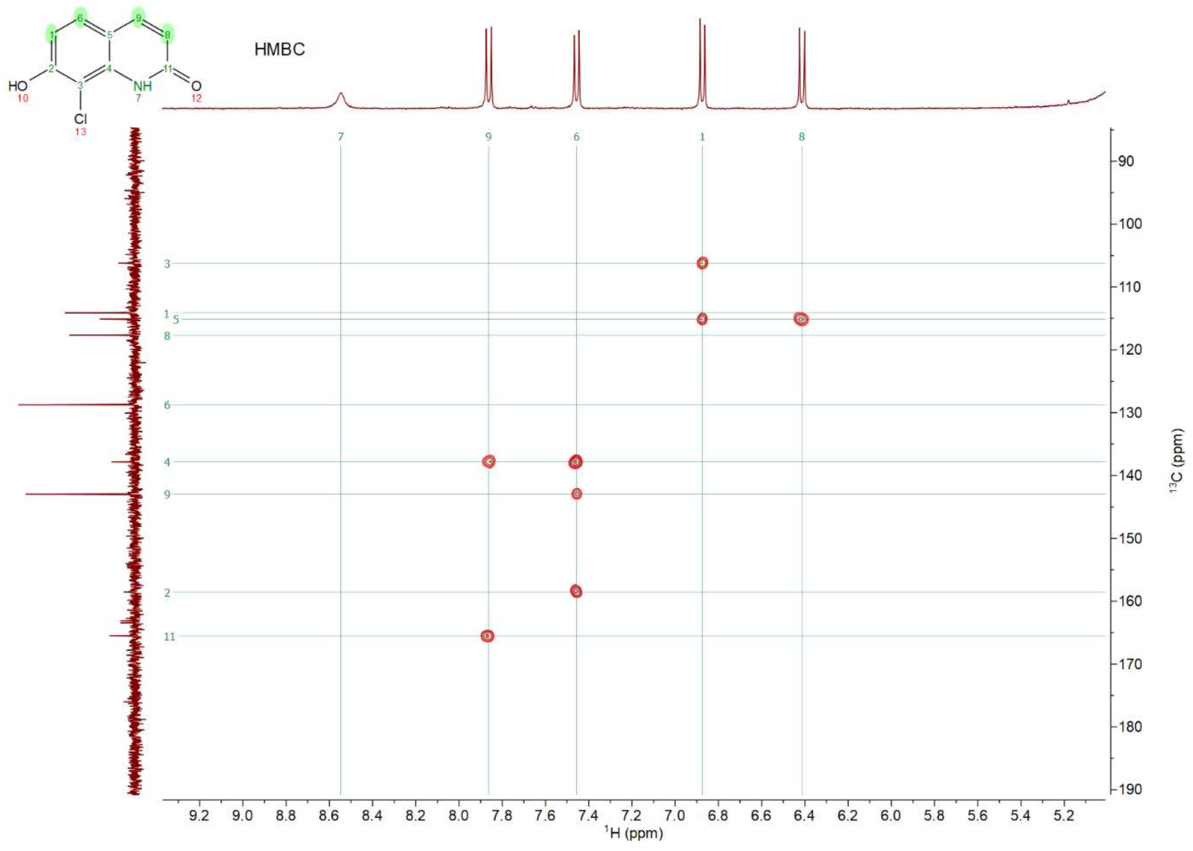

Figure S28. ^{1}H -^{13}C HMBC spectrum for 19-Cl.

22-Cl

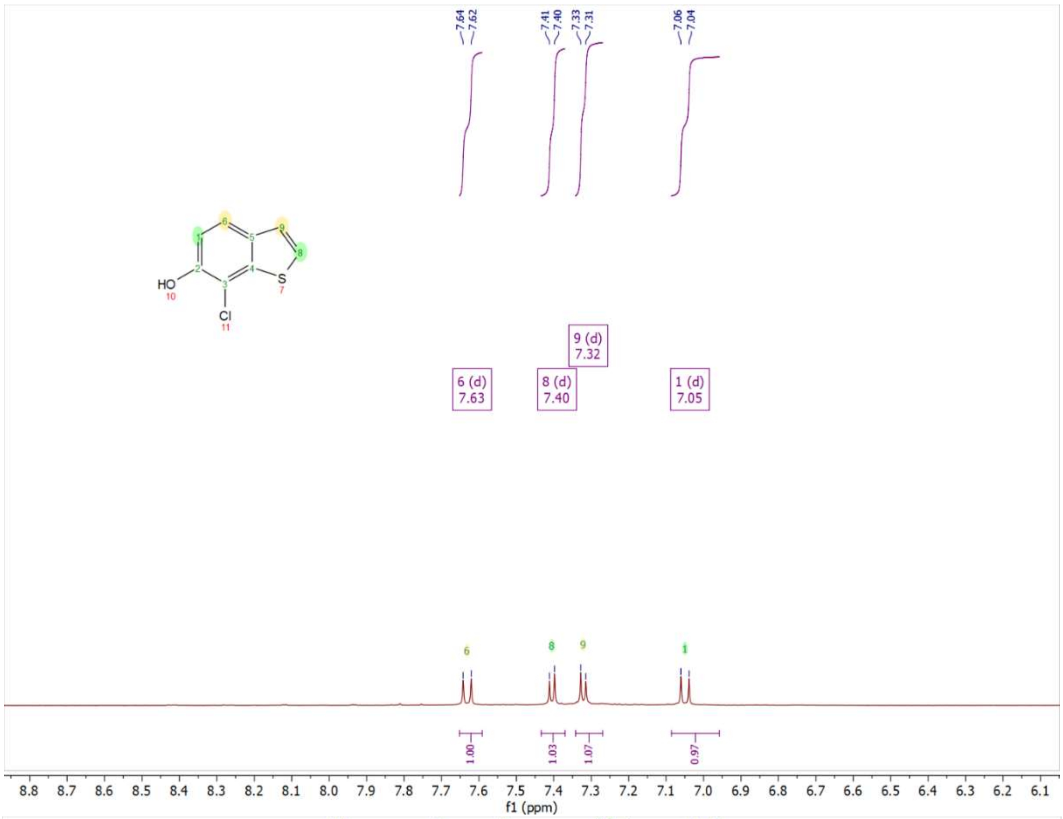

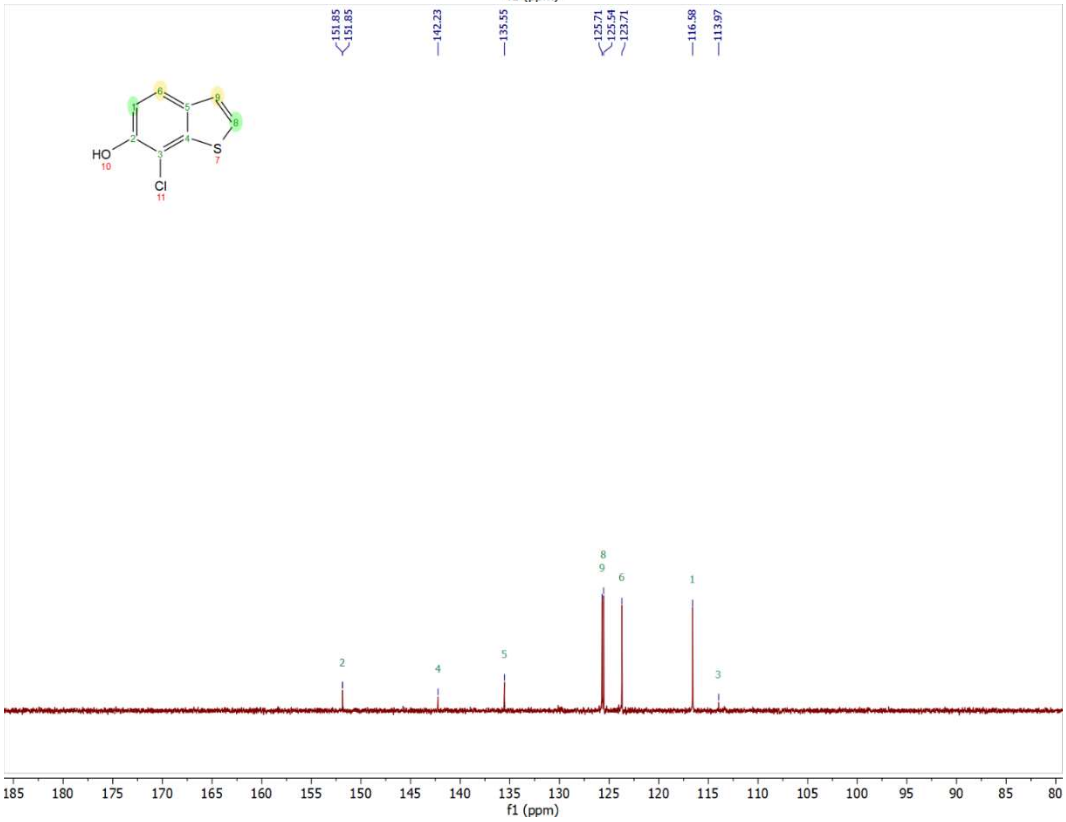

Figure S29. ^{1}H and ^{13}C NMR spectrum for 22-Cl.

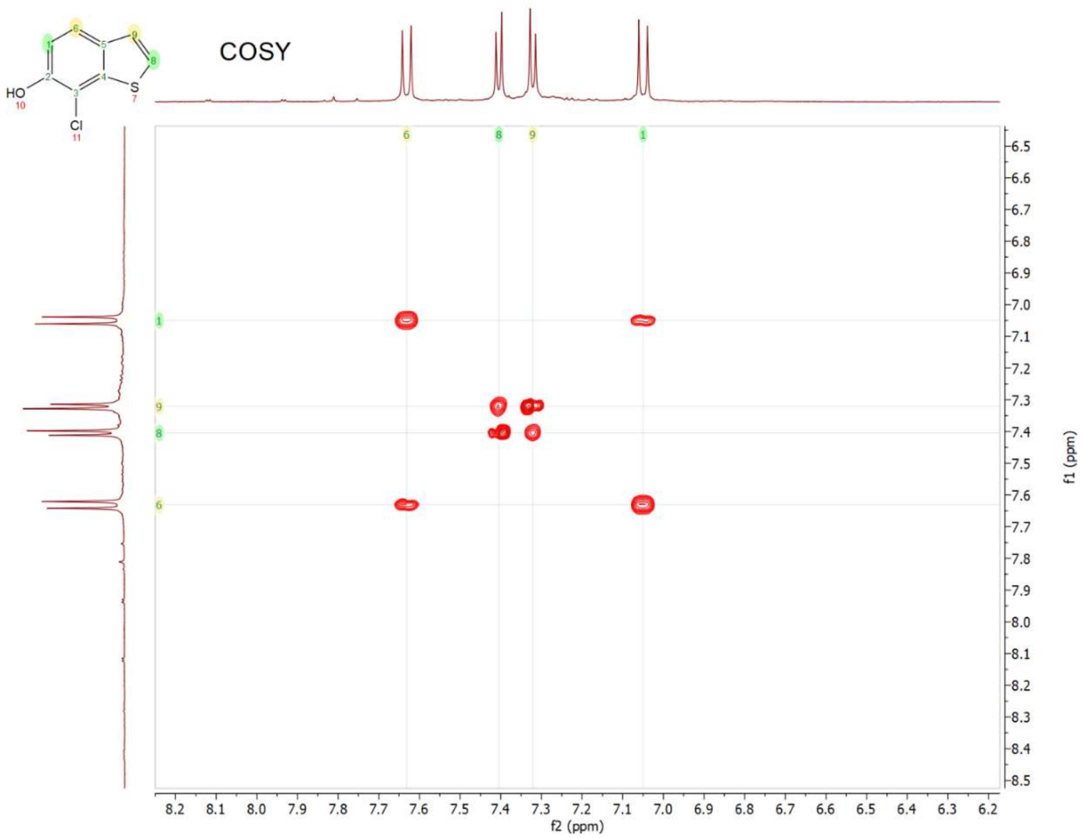

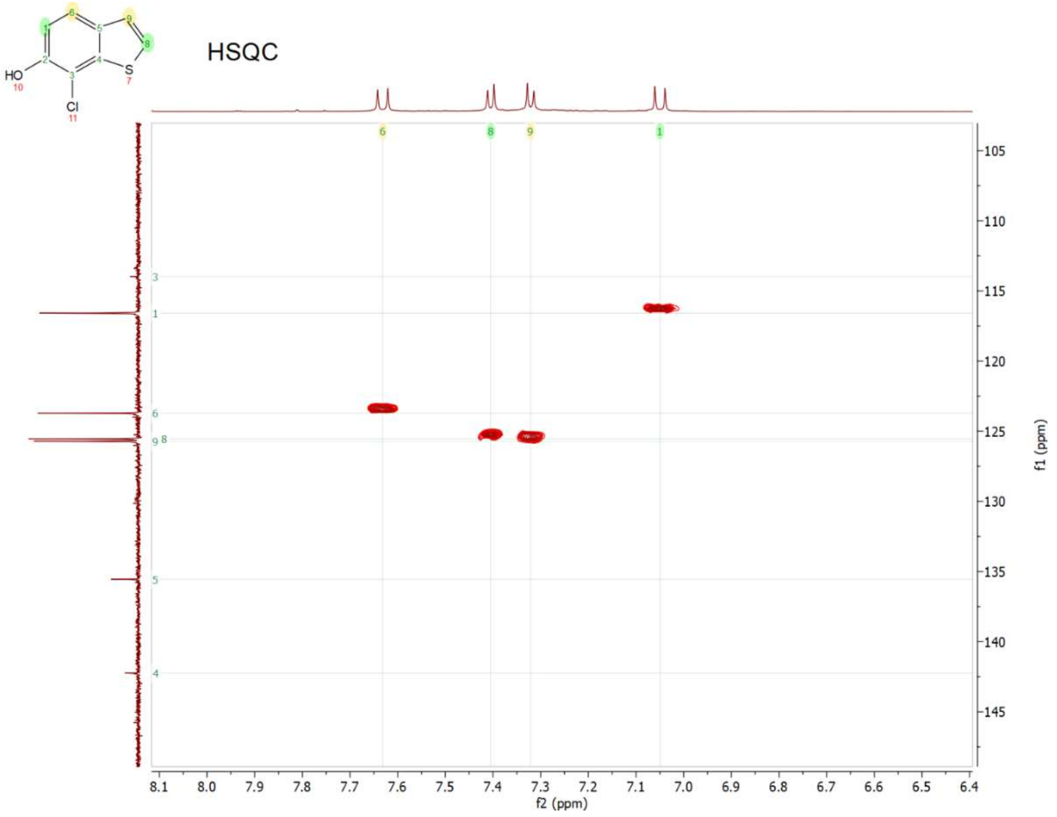

Figure S30. ^{1}H-^{1}H COSY and ^{1}H -^{13}C HSQC spectrum for 22-Cl.

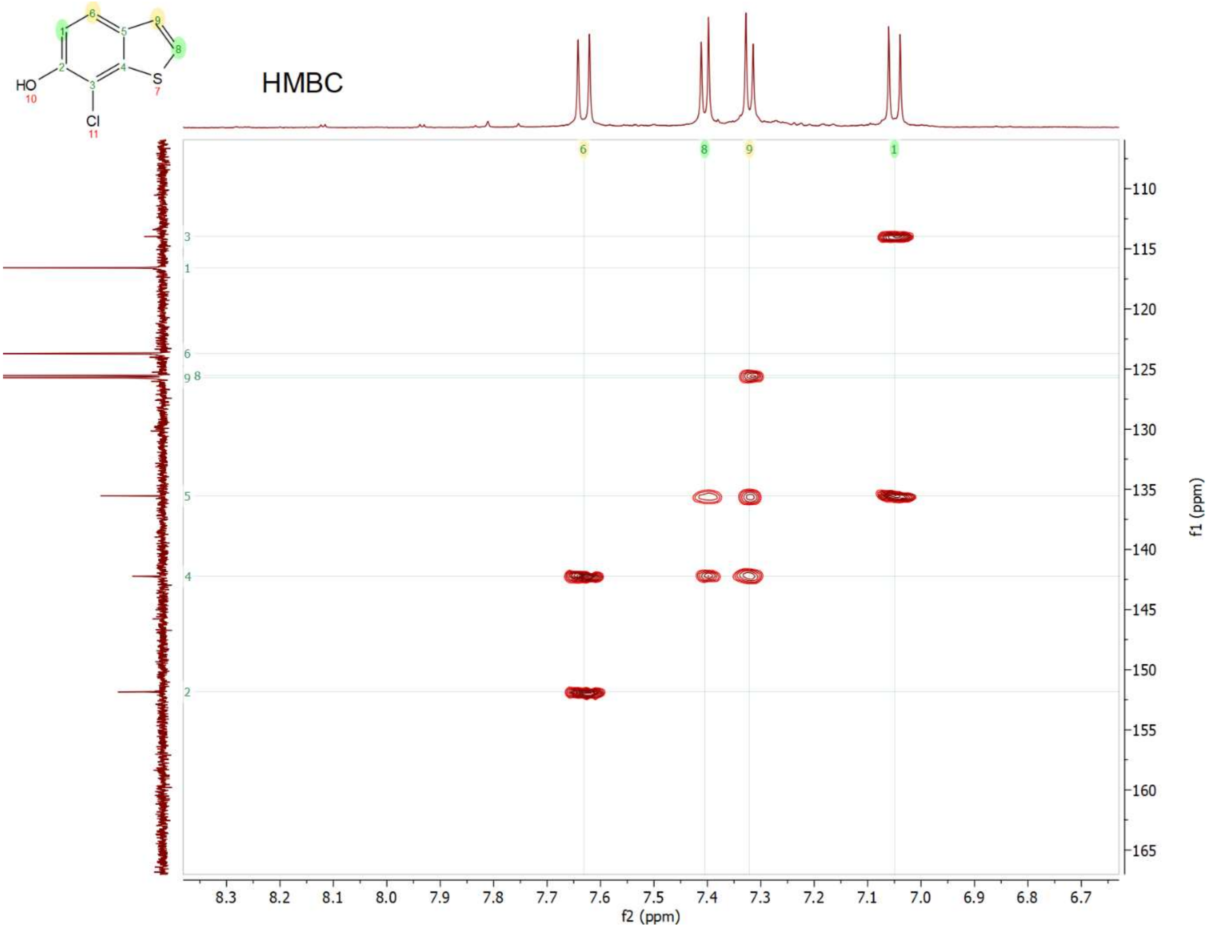

Figure S31. ^{1}H -^{13}C HMBC spectrum for 22-Cl.
